# Short chain fatty acids enriched fermentation metabolites of soluble dietary fibre from *Musa paradisiaca* drives HT29 colon cancer cells to apoptosis

**DOI:** 10.1371/journal.pone.0216604

**Published:** 2019-05-16

**Authors:** Arun K. B., Aravind Madhavan, Reshmitha T. R., Sithara Thomas, P. Nisha

**Affiliations:** 1 Agro Processing and Technology Division, National Institute for Interdisciplinary Science and Technology (CSIR-NIIST), Thiruvananthapuram, Kerala, India; 2 Microbial Processing and Technology Division, CSIR-NIIST, Thiruvananthapuram, Kerala, India; 3 Academy of Scientific and Innovative Research (AcSIR), New Delhi, India; Southern Illinois University School of Medicine, UNITED STATES

## Abstract

In this study, the prebiotic potential of soluble dietary fibre extracted from plantain inflorescence (PIF) was investigated. PIF demonstrated prebiotic potential by enhancing the growth of the probiotics under study and thereby hindered colon cancer development. The soluble dietary fibre from *Musa paradisiaca* inflorescence (PIF) was fermented using *Lactobacillus casei* and *Bifidobacterium bifidum*. The fermentation supernatants (LS and BS) were enriched with short chain fatty acids (SCFA) and were able to initiate apoptotic signalling in HT29 colon cancer cells leading to cell death. Both BS and LS exhibited cytotoxic effect; induced DNA damage and enhanced generation of reactive oxygen species in HT29 cells leading to apoptosis. The induction of apoptosis was facilitated by the reduction of membrane potential of mitochondria and ATP synthesis; enhanced delivery of cytochrome c and interference with the expression of pro/antiapoptotic proteins. BS, which exhibited better activity, was further analysed for the identification of differentially regulated proteins by performing two dimensional electrophoresis and MALDI-TOF mass spectrometry. Results emphasized on the fact that, the exposure to BSalteredthe HT29 proteins expression, particularly the upregulation of apoptosis- inducing factor-AIFM1 leading to apoptosis of HT29 cells.

## 1. Introduction

Colorectal cancer (CRC) is a leading communal health predicament, being the second most frequently identified cancer in females and the third in males globally. Increased incidence of environmental factors, in particular, lifestyle and diet alterations are reported to influence CRC epidemiology [1]. Various epidemiological studies have shown the healthy coalition between higher fibre consumption and a decreased incidence of inflammatory bowel disease and CRC [2]. Dietary fibre plays a protective role in the morbid physiology of CRC. Consumption of fibre rich grains and vegetables has been confirmed to have significant protective effect against CRC through various mechanisms [3, 4]. The main mechanism involves–reduction in transit time of faecal by enhancing the stool bulk; reduces the interaction of carcinogen with colon cells, and most importantly, the utilization of these dietary fibres by gut microbiome [5]. The human digestive system lacks enzymes that can digest dietary fibres, and these fibresare mainly fermented by colonic microbiota [6]. The gut microbiome contributes immensely to maintain a healthy intestine and alterations of this bacterial community can stimulate and lead to CRC progression [7].

The dietary fibre consumed is usually fermented by colon microbiota resulting in the production of short-chain fatty acids (SCFA) -acetate, propionate, and butyrate. These SCFA`s are endorsed with various health beneficial properties [8, 9]. Butyrate is readily absorbed by gut whereas propionate and acetate are absorbed into blood stream. Butyrate extensively nurtures colon stability and is reported for its efficacy against various diseases including cancer, chronic inflammation, diabetes etc. Butyrate induces apoptosis, and inhibits metastasis of colon cancer cells [10]; and suppresses inflammatory responses in ulcerative colitis and colon cancer [11]. Butyrate is involved in proliferation and differentiation of beta cells in pancreas and reduces diabetes associated complications [12]. Propionate lowers serum cholesterol and lipid levels [13]. Propionate and acetate reduces the risk of developing colon cancer by reverting expression of peptide YY [14, 15].

SCFA production lowers the intestinal pH which hinders pathogens and enhances the nutrient absorption [16]. *Bifidobacteria* have been reported to inhibit enteropathogens through acetate production [17]. Intestinal epithelial cells utilize butyrate as an energy source; and increases mucin production which influences bacterial adhesion [18] and enhanced tight-junctions integrity. SCFA, especially butyrate, has been studied for its part in preventing the CRC development [19]. Butyrate is known to enhance mobility of colon, decreases inflammation, induces apoptosis, and prevents the progression of tumor cells there by significantly preventing the risk of developing CRC [20]. Thus, the production of SCFA from the fermentation of dietary fibre appears to be a crucial player in the preservation of the gut health and reduces the threat of developing CRC. The activation of innate and adaptive immune systems as an outcome of microbiota disproportion usually results in chronic inflammation [21] and has been associated with an increased hazard of cancer occurrence. It has been observed that pathogens are able to endorse the inception and succession of CRC by the initiation of chronic inflammation, and hinders with the normal functioning of cell division, or origination of carcinogenic molecules that are of pro-diet origin [22].

The inflorescence from *Musa paradisiaca*, a widely cultivated plantain species in southern parts of India, has been selected for the study as there are ethno pharmacological reports that the inflorescence part serves as a remedy for gastrointestinal disorders [23]. Inflorescence is a major agro residue from plantain field as it is removed usually, at stage II, before harvesting from the stalk, for the better development of the fruits. The preliminary studies by our group reported that the inflorescence from *Musa paradisica* happens to be a rich source of dietary fibre [24]. Wehave also reported that the same is rich in polyphenols [24] and exhibits anticancer potential in HT29 colon cancer cells [25]. As the inflorescence of *Musa paradisiaca* stands rich in dietary fibre and followingcues from our earlier studies, the present study was undertaken to delineate the prebiotic properties of the dietary fibre and to investigate the anticancer potential of the metabolites produced as a result of dietary fibrefermentation by known probiotics.

## 2. Materials and methods

### 2.1 Chemicals

General cell culture reagents (DMEM, FBS, trypsin, MTT), rhodamine 123 dye, bile salts, and pepsin (Sigma Aldrich Chemicals, St Louis, USA); Antibodies were acquired from Santa Cruz Biotechnology, USA. 3–10 pH gradient IPG strips and other materials for 2D electrophoresis and western blotting were obtained from Bio Rad Laboratories (Germany). Protease inhibitor cocktail was procured from Amresco (USA). Sucrose, calcium chloride, and starch were obtained from SRL (India). Microbiological media and L-cysteine were obtained from Himedia (India) and Merck chemicals (India) respectively.

### 2.2 Cell culture

HT29 cellswas acquired from NCCS, Pune, India and retained as reported earlier (12).

### 2.3 Microorganisms

The freeze-dried cultures of *Lactobacillus casei* (NCDC17), and *Bifidobacterium bifidum* (NCDC255) were supplied by National Dairy Research Institute, Karnal, Haryana, India. *Lactobacillus casei* (NCDC17) were cultured in MRS (de Man, Rogosa and Sharpe)broth, and *Bifidobacterium bifidum* (NCDC255) were cultured in MRS broth with L-cysteine (0.05%). *Lactobacillus casei* strainswere preserved in MRS broth containing 50% glycerol at -80°C. The *Bifidobacterium bifidum* strains were maintained in MRS broth containing L-cysteine and 50% glycerol at -80°C. Each bacterial strain was grown separately and sub-cultured in fresh media before use. *E*. *coli* (MTCC 2622) was obtained from the MTCC, Chandigarh, India and cultured in the Luria-Bertani medium.

### 2.4 Extraction of dietary fibre and preparation of fermentation supernatant

Inflorescence of *Musa paradisiaca* (PI), strictly *Nendran* variety (widely used plantain variety in Kerala, India), was gathered from a local plantain field (Thiruvananthapuram, Kerala, India). The inflorescence is a large and compact structure made up of spirally arranged reddish bracts under which there are two layers of flowers. At initial stage of development each bract will turn round towards the back to expose the outer layer of female flowers that eventually develop into fruits. Later bracts will show inner layer of male flowers. Once the fruits are developed from female flowers, the inflorescence is removed from the stalk before harvesting to get better fruit yield. At this stage the whole inflorescence without any female flowers were collected (S1 Fig) and is used for the present investigation. The extraction of soluble dietary fibre (PIF) from the inflorescence was done as per the protocol described by the authors in their previous publication [24], details given in S1 File.

### 2.5 Evaluation of prebiotic properties of SDF from PI

PIF was used as an additional carbon source for the growth of the probiotic strains *Lactobacillus casei* and *Bifidobacterium bifidum*. Inoculum (0.5 mL) of the probiotic cultures were added to the media containing 1% of PIF to obtain a final concentration of 1 x 10^6^ cells/mL, mixed well and cultured for 72 h.5 mL sample was collected at an interval of 0, 24, 48 and 72 h and transferred to pre-weighed centrifuge tubes for various analyses. Inulin (1%) was used as positive control (reference standard) and the media without fibre/inulin as the control.

The prebiotic efficacy was assessed in terms of change in optical density, dry mass, decrease in pH and SCFA synthesis. After measuring the pH and OD (600 nm)at each time intervals (24 h, 48 h and 72 h), it was centrifuged at 10000 rpm for 10 min(Kubota Model No.7780, Rotor AG-6512C, Japan) and the supernatant was collected. The residue obtained was freeze-dried (VirTis genesis, USA) and the dry weight of organisms was calculated. The supernatant was analyzed for SCFA.

SCFA content in supernatant collected at different time intervals was analyzed and quantified by HPLC following the protocol of Guerrantet al. [26] with some modifications. The HPLC specifications and other details were as described earlier in our publication [24].

As the prebiotic efficacy is also correlated with improved aggregation of probiotics, inhibition of pathogenic organisms and harmful enzymes produced by them in the colon, we also evaluated the efficacy of the fermentation supernatant to promote the aggregation of the probiotics under study, inhibit the growth of *E*. *coli* and β-glucuronidase enzyme.

### 2.6 Aggregation studies

Auto-aggregation and co-aggregation studies were done according to Pan et al. [27]. The auto aggregation was calculated as

$$\%\;Auto\;Aggregation=(1-\frac{At}{A0})\times 100$$

where A_t_ represented the absorbance at time t = 1 or 5 h and A_0_ the absorbance at t = 0.

The co-aggregation assay was similar as the auto-aggregation assay. *E*. *coli* was used as a representative pathogenic strain. Equal volumes (2 mL) of the three experimental groups and *E*. *coli* were mixed in combination and vortexed for 15 s. Samples were collected similarly as that of auto-aggregation protocol and co-aggregation was determined using the formula

$$\%\;Co-aggregation=[1-\frac{\left(2Amix\right)}{\left(Aexp.+Ae.coli\right)}]\times 100$$

where A_mix_ = Absorbance of mixed cell suspension, A_exp_ = Absorbance of theexperimental group and A_e.coli_ = Absorbance of *E*. *coli*.

### 2.7 Inhibition of E. coli by fermented supernatant

The ability of fermented supernatant to inhibit *E*.*coli* was assessed by disc diffusion method. Three different volumes (10, 25 and 50 μL) of fermented supernatant from control, Inulin and PIF groups loaded in sterile paper discs were positioned on the *E*. *coli* containing nutrient agar plates; and stored at 37°C for 24 h. The diametre of zone of inhibition was taken after incubation time.

### 2.8 Inhibition of the β-glucuronidase enzyme

The ability of fermented supernatant to inhibit β-glucuronidase enzyme produced by *E*. *coli* was measuredas described by Sekikawa et al. [28].

### 2.9 MTT assay for cytotoxic activity

The cytotoxic effectof different concentrations of the fermentation supernatant (obtained at 24 h) was performed in HT29 cell lines following the procedure mentioned in our previous publication [25].

### 2.10 Hoechst 33258 staining

Nuclear condensation or fragmentation happens to be one of the hallmarks of Apoptosis and this was visualized by Hoechst 33258 staining. The impact of fermentation supernatant on nuclear fragmentation of HT29 cells were measured by the method described by Harada et al. [29].

### 2.11 Apoptosis assay by flow cytometry

We performed flow cytometry to examine the initiation of apoptosis by fermentation supernatant on HT29 colon cancer cells. The assay was performed using an apoptosis detection kit (Cayman Chemical Company, USA) [25].

### 2.12 Intracellular reactive oxygen production

The generation of intracellular reactive oxygen species was validated using the DCFH-DA fluorescent method [30].

### 2.13 Evaluation of mitochondrial membrane potential

Fluorescent dye Rhodamine 123 (Rh123) was employed for determining the impact of fermentation metabolites on the membrane potential of mitochondria of the HT29 cells. Flow cytometry was carried out to investigate whether the fermentation supernatant could decrease the mitochondrial membrane potential following the protocol described in our preceding report [25].

### 2.14 Estimation of ATP synthesis by HPLC

HPLC method was adopted [31] with slight modifications [25] to study the effect of fermentation supernatant on ATP synthesis.

### 2.15 Detection of release of cytochrome C

The release of cytochrome C was determined as described in previous reports[25, 32].

### 2.16 Apototic/antiapototic protein detection by western blot

The expression of major *apototic/antiapototic* proteinswas performed according to the illustrated procedure from our earlier publication[25].

### 2.17 Proteome analysis by 2D electrophoresis

HT29 cells after the treatment with fermentation supernatant was analyzed for the expression of various proteins by performing two-dimensional electrophoresis. The detailed procedure is depicted in our previous report [25].

### 2.18 Statistical analysis

All data showed in this research article were expressed as mean ± standard deviation of triplicate measurements. The results were tested with one way analysis of variance (ANOVA) and the significance of differences between means were calculated by Duncan’s multiple range test. The statistical comparisons were performed using SPSS (standard version 7.5.1, SPSS Inc., USA). p ≤0.05 was appraised as statistically significant.

## 3. Results and discussion

### 3.1 Prebiotic efficacy of dietary fibre

The plantain inflorescence has been reported to contain 12.45 ± 1.14% (DW) of double dietary fibre [24]. The prebiotic potential of the PIF has been evaluatedin terms of its potential to support the growth of two well-known probiotic species–*Lactobacillus casei* and *Bifidobacterium bifidum* when incorporated in the growth media. This was assessed in terms of lowering pH, increase in optical density, dry mass and production of SCFA. As can be seen (S2A–S2C Fig), the pH of the medium supplemented with the dietary fibres (PIF and inulin) decreased relevantly in comparison to the control indicating the utilization of this fibre by the organisms for their growth. The change in the pH of the media with PIF was significantly higherthan that of the media with inulin. In the case of *L*. *casei* and *B*. *bifidum*, the pH of the media with PIF decreased to 4.06 and 4.0 after 72 h of fermentation, from an initial pH of 5.905 and 5.75, respectively, whereas the same for inulin was 5.955 to 4.345 and 5.88 to 4.285 respectively (S2A Fig). The turbidity measurement of the growth medium at 600 nm can be related to the growth of microorganisms indirectly. It was found that the optical density of the media with PIF was significantly higher than that of the inulin which indicates that PIF support the growth of probiotics (S2B Fig). The increase in the dry mass of the PIF incorporated media was considerably higher than the positive control and the control media (S2C Fig). The pH, OD, dry mass and colony count measurement indicates that SDF from PI promotes the growth of probiotic bacteria which was significantly higher than the inulin and control group. The SCFAs in experimental groups at different time intervals of incubation were identified and quantified by comparing with standards (S3 Fig and S1 Table). It could be seen that SCFA production increased when PIF was added to the growth media, both in the case of *L*. *casei* and *B*. *bifidum*. Butyric acid production by *L*. *casei* after 72 h incubation time in the media with PI and inulin were 37.60 and 29.28 μg/mL respectively. The same for *B*. *bifidum* was 45.60 and 37.28 μg/mL, respectively. As observed, *B*. *bifidum* produced maximum butyric acid which resulted in corresponding decrease in the pH of the mediain correlation to SCFA production.

Probiotic microorganisms restore the balance of the gut flora, while prebiotics provide energy for these beneficial bacteria. In combination, they sustain the proliferation of vital bacteria in our bowels and encourage their activity and chances of survival. Dietary fibre can be termed as a prebiotic only if it could alter the intestinal microbiome towards a better configuration by controlling the adverse pathogens, and in turn stand favourable to gut health [33]. The optical density, pH and dry mass showed that the selected probiotic species–*Lactobacillus casei* and *Bifidobacterium bifidum* grows better in the media containing PIF than the media without PIF and positive control inulin. The optical density and dry mass showed direct correlation with the bacterial mass in the media [34] that increased significantly in the sample in comparison with the positive control, in the present study. The decrease in the pH value could be connected to the SCFAs, as they are important metabolites formed during the fermentation of prebiotic by probiotics [20]. As the number of viable organism increases, further will be the production of SCFA which lower the pH. Probiotic strains metabolize undigested carbohydrates to reclaim energy which in turn produces acetate, propionate and butyrate [20] that exert positive effects on human health by various mechanisms [35]. Acetate, propionate and butyrate are the important SCFAs and butyrate being the most important one [9]. The HPLC analysis showed that the production of all the three SCFAs in the media incorporated with PIF increases significantly, after the incubation period.

#### 3.1.1 Aggregation studies

Bacterial auto-aggregation and co-aggregation properties help in maintaining the gut homeostasis by colonization of the probiotic strains that help in excluding the pathogens. In our study, we performed this experiment to prove that thePIF enhances the growth of the probiotics and hence they have better aggregation properties when compared to other experimental groups. The results from the study indicated that the auto and co-aggregation properties of *L*. *casei* and *B*. *bifidum* increases when PIF was included in the growth media.

**Fig 1:**
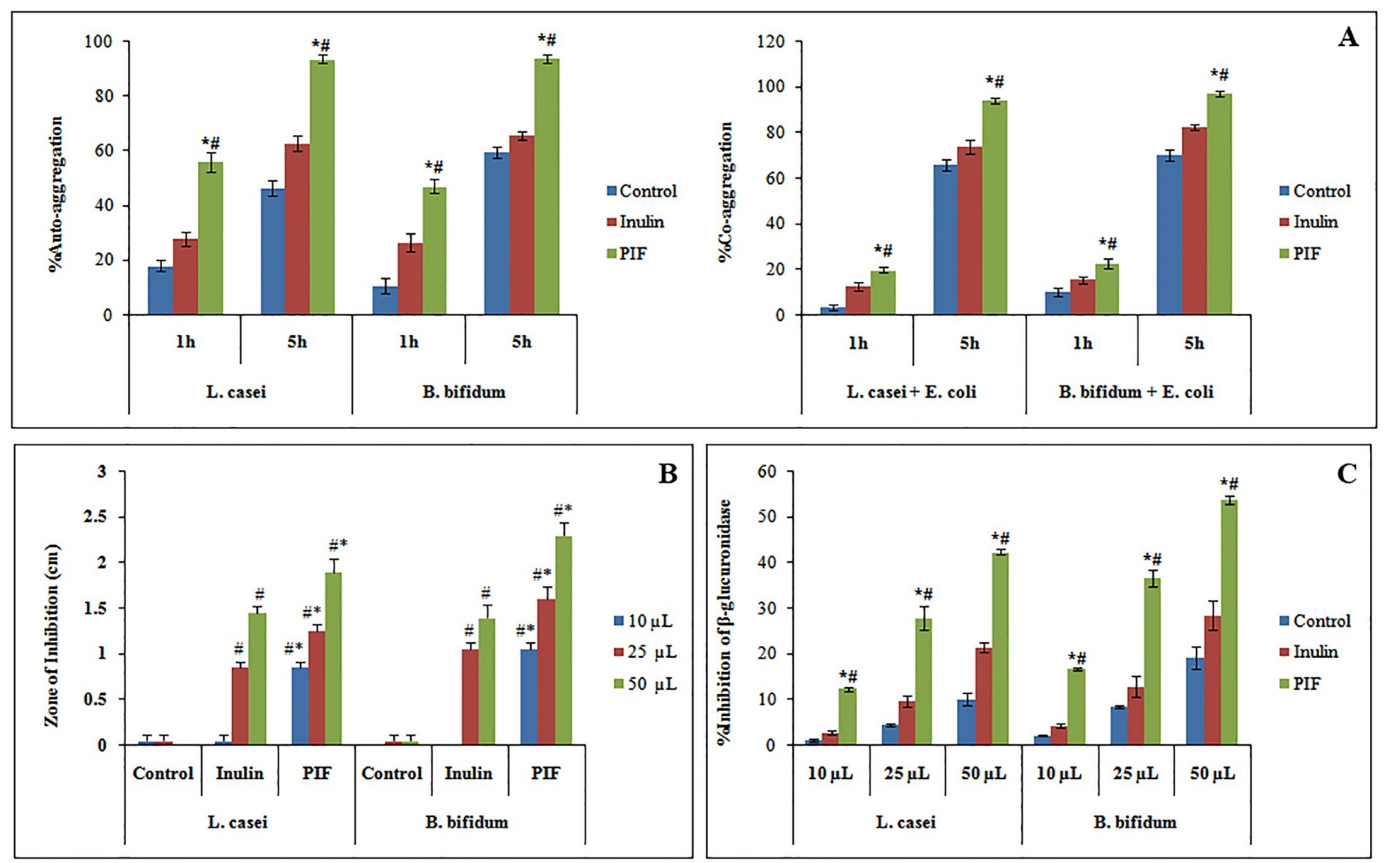
**[A]** Auto and Co-aggregation efficacy of *L*. *casei* and *B*. *bifidum*. **[B]** Graphical representation of antibacterial activity of fermentation supernatant from different experimental groups against *E*. *coli*.**[C]** βglucuronidase inhibition by fermentation supernatant LS and BS. Values plotted are average of triplicate experiments. ^#,^*Correspondingly denotes significant difference of PIF from control and Inulin group. p ≤0.05 was considered statistically significant.

After 5 h incubation, the auto-aggregation increased from 56 to 93.6% and 47.05 to 93.66% respectively for *L*. *casei* and *B*. *bifidum* in the medium incorporated with PIF which was significantly better than the inulin incorporated media (Fig 1A). Auto-aggregation triggers the development of biofilm in the gastrointestinal tract which preserves the intestine by forming a barrier and enhancing the immunity [36], and prevents colonization of harmful bacteria [37]. Moreover, the high auto-aggregation potential of probiotic strains helps them to colonize effortlessly in the intestinal walls [37]. This will inhibit the adhesion of pathogens and hence prevent pathogen translocation and subsequent infection [38]. The co-aggregation percentage with pathogenic *E*. *coli* increases from 19.85 to 94.2% and 22.75 to 97.1% respectively for *L*. *casei* and *B*. *bifidum* incorporated with PIF after 5 h incubation, which is better than Inulin included media (Fig 1A). The efficacy of antimicrobial metabolites synthesised by live probiotic happensto be enhanced as co-aggregation minimizes the intercellualr distances between probiotics and pathogens[38]. Even though the aggregation properties are highly strain dependent, the presence of prebiotics multiplies the probiotic number which indirectly enhances the aggregation properties.

#### 3.1.2 Inhibition of E. coli by fermented supernatant

One of the important mechanism by which the probiotics promote gastro-intestinal health is by preventing the growth of pathogenic strains—by the antimicrobial action of secondary metabolites produced by fermenting undigested carbohydrates. Hence we analyzed the antimicrobial effect of supernatant from the fermentation of PIF on the growth of *E*. *coli*.

From the results (Fig 1B, S4 Fig) it was evident that the fermentation supernatant obtained from the media with PIF inhibited *E*. *Coli* much effectively than that of the known prebiotic Inulin. For 50 μL fermentation supernatant with PIF, the zone of inhibition was 1.9 ± 0.141 and 2.3 ± 0.141 cm for *L*. *casei* and *B*. *bifidum*, respectively. However, the corresponding zone of inhibition for Inulin group (50 μL) was found to be only 1.45 ± 0.07 and 1.4 ± 0.141 cm. The zone of inhibition was found to increase with the amount of supernatant which possibly could be correlated to the increase in the amount of short chain fatty acid present in the supernatant.

#### 3.1.3 Inhibition of the β-glucuronidase enzyme

Reddy and Wynder [39] had correlated high content of β-glucuronidase with increased colon cancer risk and one of the primary factors in the etiology of colon cancer. Formation of β-glucuronidase and its activity is reduced by high-fiber diet, probiotics, and low meat diet that lower the colonic pH. Therefore, in the present study, we analyzed the effect of fermented supernatant (10, 25 and 50 μL) of different experimental groups, in inhibiting β-glucuronidase produced by *E*. *coli*. The inhibition of β-glucuronidase (%) for the supernatant (50 μL) from PIF and inulinwas found to be 42.36 and 21.55 for *L*. *casei*, and 53.78 and 28.55 for *B*. *Bifidum* respectively (Fig 1C) which demonstrate the potential of PIF in the enzyme inhibition.

From the above studies, it has been established that the soluble dietary fibre from plantain inflorescence exhibits significant prebiotic potential as it effectively promotes the growth of selected probiotic strains (*L*. *casei* and *B*. *bifidum)* and enhances the production of SCFA in the corresponding fermentation medium. The inhibition of the growth and β-glucuronidase production by *E*. *Coli*s shown in the present studystands very crucial in maintaining the homeostasis of the colon. The SCFA has been reported to exhibit anticancer potential especially against CRC [40]. Since SCFA is one of the important secondary metabolitewhich was found to be increased in fibre incorporated medium, we were interested to study further the anticancer effect of fermentation supernatant from PI (PIF) and the possible mechanism behind it. Hence the anticancer potential of fermented supernatant (after 72 h incubation) was investigated for its anticancer activity on HT29 colon cancer cells.

### 3.2 Anticancer potential of PIF fermentation supernatant containing SCFA

#### 3.2.1 Cytotoxic effect of PIF fermentation supernatant

**Fig 2:**
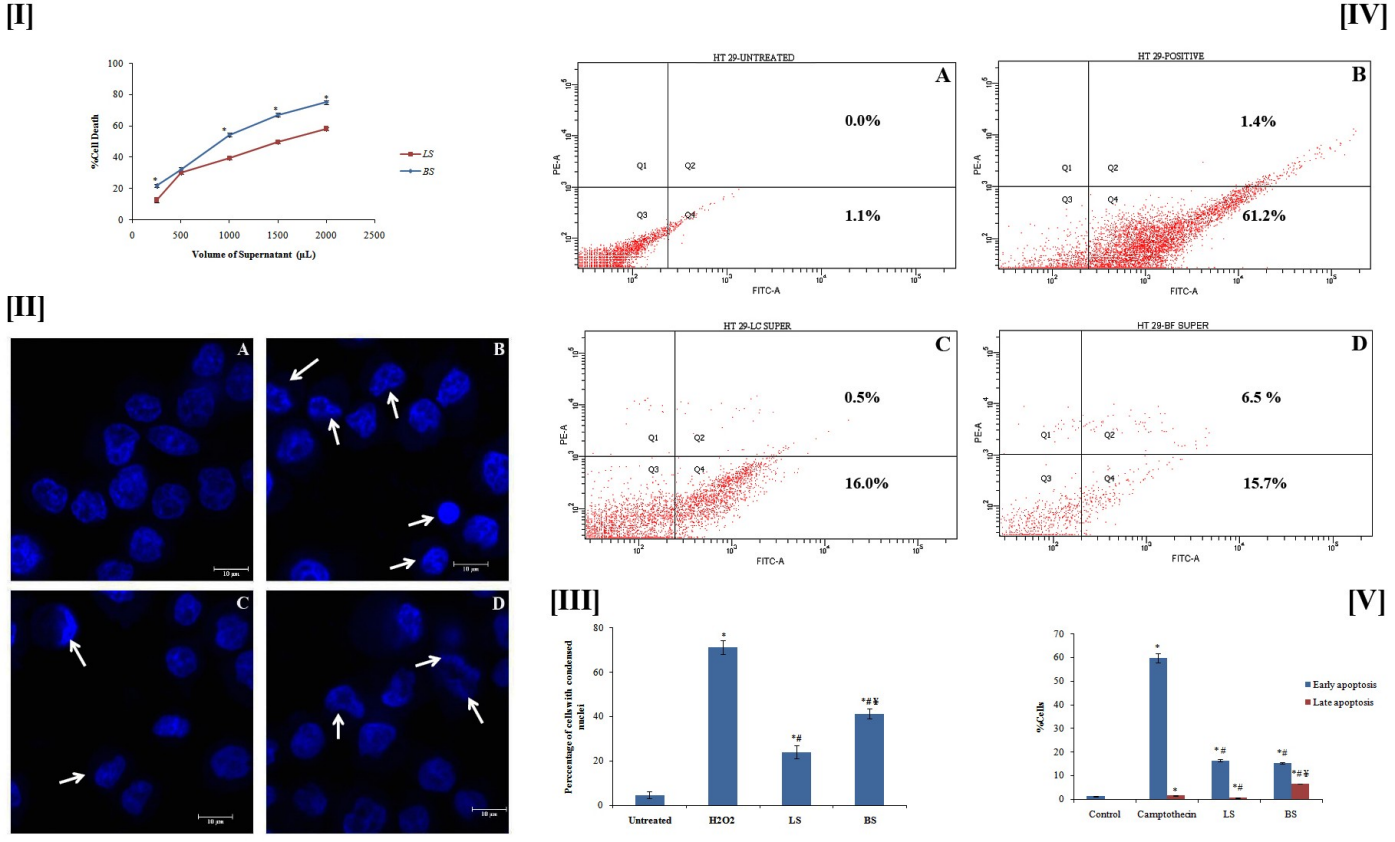
**[I]** Cytotoxicity of fermentation supernatant LS and BS. Each value represents the mean ± SD from triplicate measurements. *BS significantly different from that of LS. **[II]** Observation of DNA condensation (denoted by arrows) by Hoechst staining: Figure shows (A) control, (B) H_2_O_2_ (250 μm), (C) LS and (D) BS.**[III]** Graphical representation of percentage of cells with condensed nuclei. **[IV]** Flow cytometry data forapoptosis induction. Figure displays (A) control, (B) Camptothecin, (C) LS and (D) BS. Quadrants Q1to Q4 correspondingly depicts dead, lateapoptotic, live, and early apoptotic cells. **[V]** Graphical representation of percentage of cells in early and late apoptosis stage. Values plotted in III & V are average from triplicate measurements. *^,#^Correspondingly designates significant change from the untreated and positive control group. ^¥^ BS significantly different from LS. # p ≤0.05 was considered statistically significant.

The anticancer studies were carried out using the fermentation supernatant after 24 h of incubation. It was foundfrom the MTT assay that the fermentation supernatant exhibited cytotoxicity (Fig 2[I]). The IC_50_ value for the fermentation supernatant of *L*. *casei* with PIF (LS) was found to be 1510.88 μL, whereas the same for *B*. *bifidum* (BS) was 905.75 μL. BS exhibited better cytotoxic effect, may bedue to thepresence of higher content of SCFAs.500 μL of LS and BS were used for further studies (concentration bellow IC_50_ value). The HPLC quantification of SCFAindicated the presence of 27.265 μg of acetic acid, 15.65 μg of propionic acid and 10.66 μg of butyric acid in 500 μL of LS whereas the same for BS were 30.925, 19.785 and 15.155 μg respectively. Thehigher concentration SCFA in BS may be correlated with better utilization of PIF by *Bifidobacterium bifidum*. H_2_O_2_-250 μM was employed as positive control. The cytotoxicity of SCFAs on colon cancer cells has been reported earlier [41].

#### 3.2.2 Fermentation metabolite induces DNA condensation

The DNA condensation/fragmentation is a trademark of cells moving towards apoptosis [42]. Apoptotic cells are characterised with compressed and fragmented DNA [43]. The effect of LS and BS on the nuclear changes of HT29 colon cancer cells was studied by the DNA-binding fluorescent dye (Hoechst 33342 stain). As shown inFig 2[II], cells treated for 24 h with LS, BS, and H_2_O_2_ showed notable changes in nuclear structure when compared to the untreated sample. In contrast, the untreated cells remained uniformly stained. BS was found to induce more DNA condensation as compared to LS. The morphological analysis indicated that LS and BS induced DNA condensation HT29 cells which is an indication of apoptosis in. However, the activity of the supernatant was less when compared to the positive control (H_2_O_2_). We counted number of cells with condensed nuclei in ten different fields and the average percentage was plotted (Fig 2[III]). The results showed that BS induced condensation in 41.33 ± 2.08% cells which is significantly better than LS (24 ± 3%).

#### 3.2.3. Apoptosis induction by fermentation supernatant

Apoptosis is a tightly controlled programmed cell death contributing to the exclusion of unwanted cells in order to retain the equilibrium between survival and death of cells. In the incidence of cancer, apoptosis fails to ensue which results in generation of malignant cells that are immortal. The apoptosis system is multifaceted and abnormalities in apoptosis play important roles in tumor pathogenesis. Therefore, cancer research essentially concentrates in the induction of apoptosis in cancer cells.

Since LS and BS demonstrated cytotoxicity and DNA condensation effect, its ability to induce apoptosis was furtherinvestigated. As can be seen from the flow cytometrystudies (Fig 2[IV] and 2[V]), it was clear that the BS had a better effect in inducing apoptosis than LS, particularly in late apoptosis stage. But, the activity is not significant when compared with standard camptothecin as it has better activity than LS and BS, particularly in early apoptosis stage. However, these results confirmed that the supernatants, obtained from fermentation of PIF by the probiotic bacteria, induce apoptosis in HT29 cells. These experimental outcomes are in line with the earlier reports explaining the apoptotic effect of fermentation supernatant from various sources [44, 45].

#### 3.2.4 Determination of intracellular reactive oxygen production

**Fig 3:**
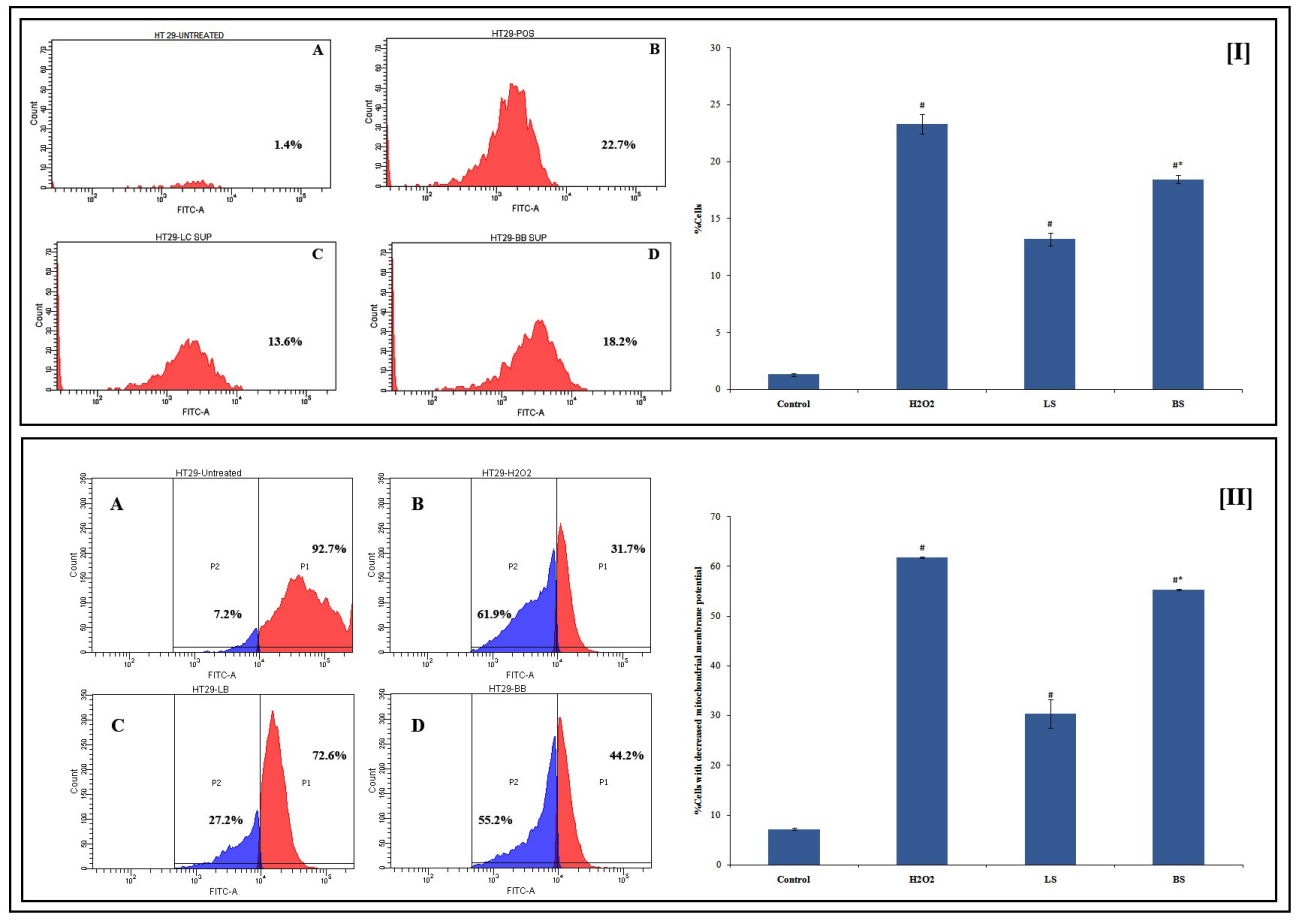
Flow cytometry data and graphical representation showing impact of fermentation supernatant LS and BS on [I]ROS production and [II] mitochondrial membrane potential. The values are avverageof triplicate measurements. ^#^Significantly different from the control. *BS shows significant change from LS. A-D correspondingly represents untreated, H_2_O_2_, LS, and BS.p ≤0.05 was considered statistically significant.

The apoptotic inducing capacity of the LS and BS prompted us to investigate further on its mechanism. A variety of stimuli can initiate apoptosis. ROS and oxidative damage have been associated in the signalling of apoptosis. Hence we analyzed whether ROS production showed increase after the treatment by supernatant in HT29 cells. The level of reactive oxygen species in HT-29 cells was estimated using DCFH-DA using flow cytometry. The comparison with untreated control cells showed that the ROS levels in treated HT-29 cells increased significantly. Among the supernatant, BS was found to induce more ROS production than LS (Fig 3I). Various anticancer drugs are known to upregulate ROS production in cancer cells and induce apoptosis [46]. Butyrate is reported to trigger apoptosis in HT29 cells [47]. Hence the apoptotic effect of fermentation supernatant may be related to the presence of butyrate. The increased production of ROS in cells pre-treated with BS leading to apoptosis may be linked to the higher butyrate content in BS.

#### 3.2.5 Mitochondrial membrane potential (Δψ_m_) was decreased

The Δψ_m_ of cancer cells are more hyperpolarised than normal cells. The Δψ_m_ is reported to have a great influence in the genesis of colon cancer. The elevation in the mitochondrial membrane as well as the structural abnormalities and function of the membrane has been related to colon carcinoma [48]. During apoptosis the proapoptotic BAX and/or BAK translocation from the cytosol to the mitochondria which results in dissipation of Δψ_m,_ further releasing cytochrome c and other elements [49]. These events drive the recruitment and processing of pro-caspase-9, leading to terminal apoptosis [50]. Degeneration of Δψ_m_ has been reported to decrease or inhibit apoptosis that can be correlated with the elevated mitochondrial membrane potential levels in colonic tumors [51].

The histograms obtained from flow cytometry analysis showed that the Δψ_m_wasreduced when treated with LS and BS. The activity of fermentation supernatant was less when compared to the positive control (H_2_O_2_) used in this assay. It was found that the loss of mitochondrial membrane potential was 61.8 ± 0.1% in H_2_O_2_ (250 μM) treated cells whereas the same for LS and BS treatment were 30.37 ± 2.84 and 55.27 ± 0.115% respectively (Fig 3II). Thereduction in the Δψ_m_ as evident from current experiment remains interesting, as the production of ATP is linked to mitochondrial membrane potential and thus can lead to initiation of apoptic events e.g., release of Cytochrome c.

#### 3.2.6 Determination of ATP production

Cancer cells divides uncontrollably and therefore have high demand for ATP as it is the main energy bank for the cells. ATP plays vital role in the processes that mediates all types of cell death–apoptosis, autophagy, and necrosis. Late-stage apoptosis is associated with decreased levels of ATP due to loss of mitochondrial function and consumption by ATP-dependent proteases. There for an increased production of ATP is essential for the persistence of cancer cells. In this perspective, one of the focuses of anticancer studies could be to evaluate the production of ATP by the cells.

**Fig 4:**
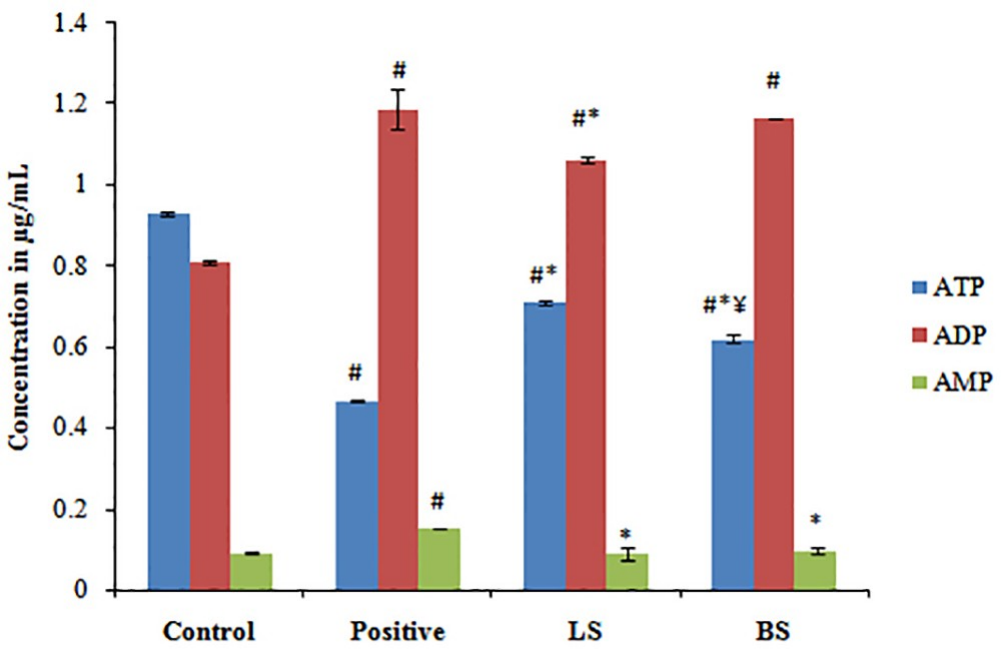
Effect of fermentation supernatant LS and BS on ATP synthesis in HT29 cells. ATP, ADP and AMP content of untreated, positive control, LS and BS treated cell sare plotted. The values denotes average of triplicate measurements. ^#,^*^,^Correspondingly shows significant difference from control and positive group. ^¥^BS shows significant change from LS. p ≤0.05 was considered statistically significant.

The ATP synthesis in HT29 cell lines after incubation with fermentation supernatant wasanalysed by HPLC as reported earlier [25]. The results pointed out that the ATP synthesis was diminished after the treatment of HT29 colon cancer cells with LS and BS. The control group was estimated to have an ATP content of 0.929 ± 0.004 μg/mL, which was abridged to 0.4675 ± 0.002 μg/mL, when treated with H_2_O_2_. It can be noted that the ATP production when treated with LS and BS were 0.709 ± 0.033 μg/mL and 0.619 ± 0.031 μg/mL respectively (Fig 4). The decreased production of ATP cells treated with BS and LS, in comparison with the control cells could be correlated with the loss of mitochondrial potential. The production of ATP is influenced by the enzymes of the glycolytic pathway. Therefore, the reduction in ATP production may also be due to the interaction of fermentation supernatant with these enzymes. The results demonstrated that BS has better activity than LS in reducing the ATP production.

#### 3.2.7 Determination of cytochrome C release

The cytochrome c releasefrom mitochondria is the initial foot step of apoptosis. Thecytochrome cgetsfixedtoAPAF-1 protein further activates caspases that are crucial for the execution of apoptosis. The antiapoptoticBCL2 proteins are usually overexpressed in cancers including colorectal cancer [52], which prevents cytochrome c release and thereby preventing the cells from entering apoptosis.

**Fig 5:**
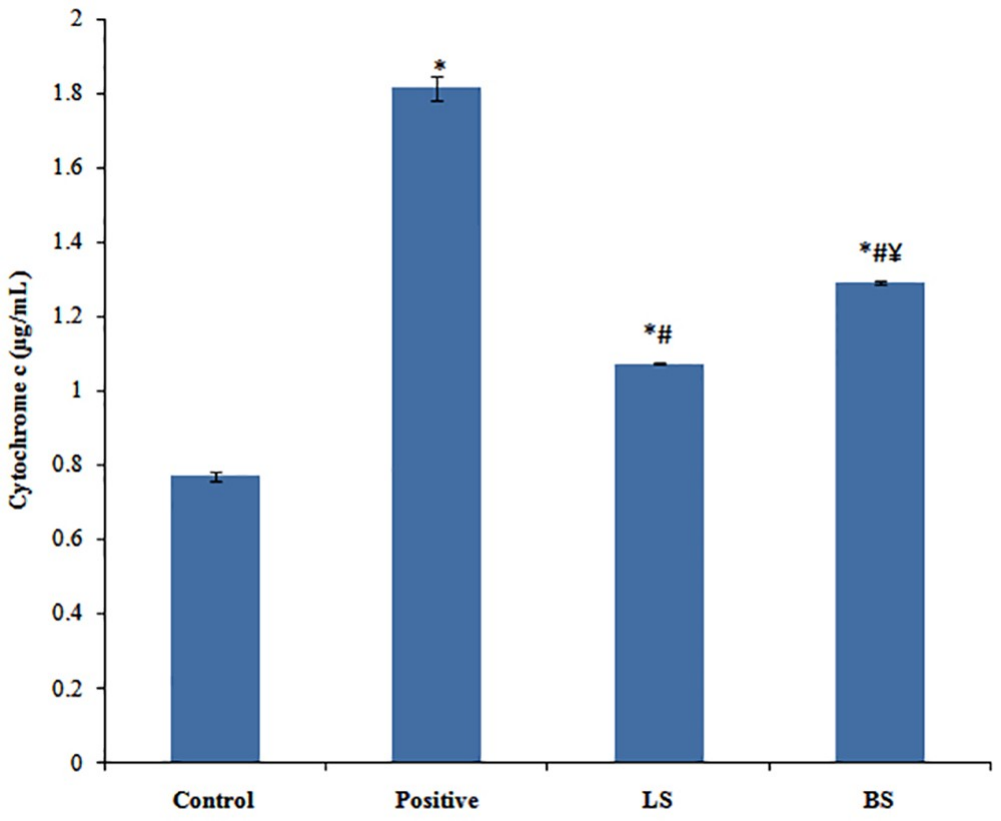
Histogram showing concentration of cytochrome c released from untreated, positive control, LS and BS experimental group. The values depicts average of triplicate measurements. *^,#^Correspondingly shows significant difference from control and Inulin group. ^¥^BS shows significant change from LS.p ≤0.05 was considered statistically significant.

Hence we analyzed the effect of fermentation supernatant LS and BS on cytochrome c release by HPLC as reported earlier [25]. As can be noted, the release of cytochrome c was enhanced in experimental groups [1.074 ± 0.023 μg/mL(LS) and 1.292 ± 0.027 μg/mL (BS)] when compared to the untreated cells (0.771 ± 0.012 μg/mL) (Fig 5). The same for the positive control was 1.813 ± 0.031 μg/mL. The activity of BS was significantly better than LS.

#### 3.2.8 Western blot analysis

The results from the above assays evince that the fermentation supernatants, LS and BS, have significant anticancer effect on HT29 colon cancer cells by inducing apoptosis. To ascertain this, we analyzed the expression of some of the key proteins involved in the apoptosis after treating the HT29 cells with LS and BS.

The BCL-2 protein family consist of anti/pro-apoptotic proteins that mainly control the events of apoptosis. The apoptotic process is inhibited by the anti-apoptotic members of this group, such as BCL2, either by averting the release of cytochrome c or by sequestering the caspases. On the contrary, pro-apoptotic members, such as BAX, elicit the release of cytochrome c and leads to the caspase activation. The cytochrome c initiates breaking of caspases which leads to the cleavage of poly(ADP-ribose) polymerase (PARP) and cleaved PARP is mainly assayed as a marker for apoptosis [53].

**Fig 6:**
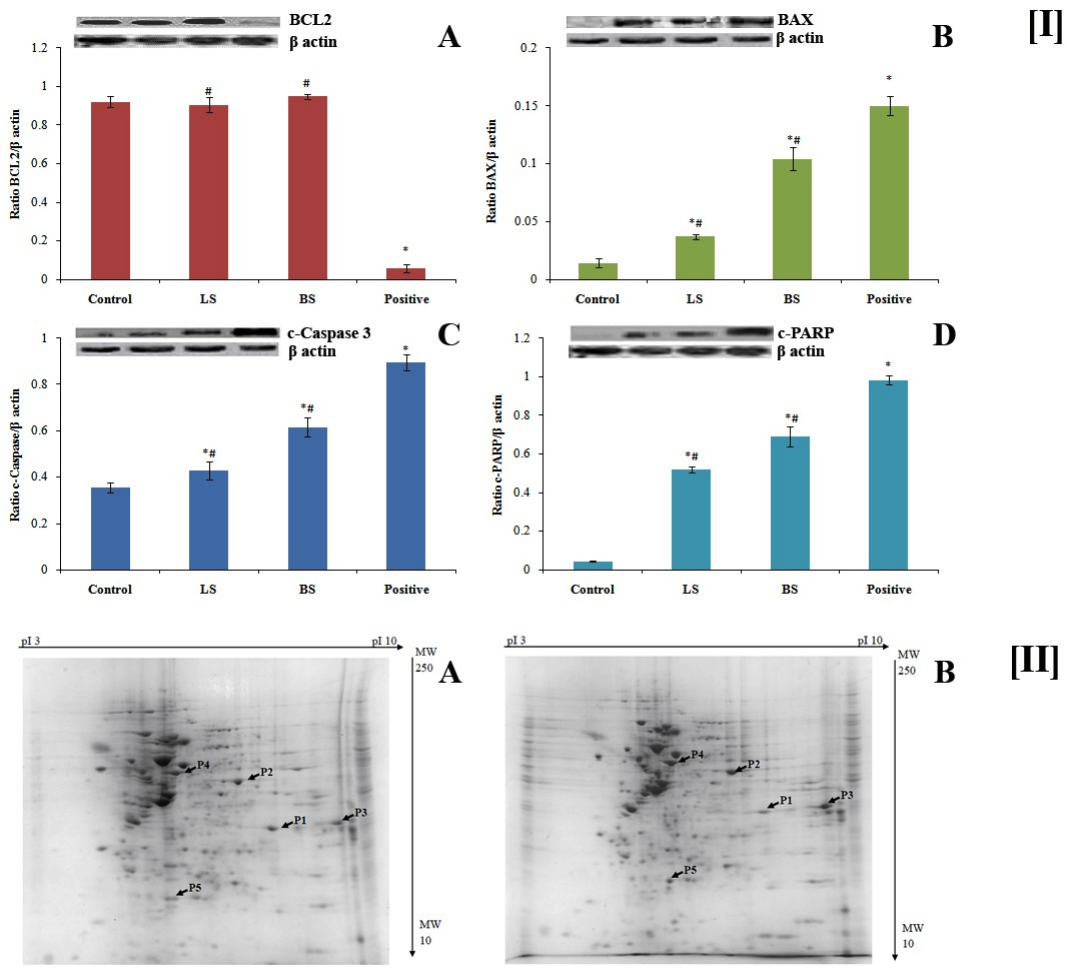
**[I]** Effect of fermentation supernatant LS and BS on the expression of apoptotic proteins. Protein from untreated, LS, BS and 5-Fluorouracilexperimental groupswere loaded in lanes 1–4 respectively. A-D correspondingly shows BCL2, BAX, c-caspase 3 and c-PARP. The values are average of three different readings. * ^and #^ Significantly different with respect to control and positive group respectively. p ≤0.05 was considered statistically significant.**[II]** Two dimensional profile of total protein from untreated (A) and BS treated (B) HT-29 cells. (B) BS. The identified proteins are indicated by P1 to P5.

We have analyzed the expression levels of cleaved PARP (c-PARP), cleaved caspase (c-caspase), BCL2 and BAX after the treatment of HT29 cells with LS and BS. The results suggested that LS and BS do not affect BCL2 expression (Fig 6IA). However, pro-apoptotic protein BAX showed an increased expression after the treatment with LS and BS (Fig 6IB) which was higher for BS as compared to LS. The fermentation supernatant LS and BS were able to manifest the establishment of c-caspase 3 as well as c-PARP (Fig 6IC and 6ID). Therefore, it may be hypothesized that the activation of BAX initiated the release of cytochrome c which in turn lead to breaking of caspase 3 and PARP that sequentially initiated the cell death. Both LS and BS were less active compared to 5-Fluorouracil (50 μM), the standard used in the assay.

The fermentation of dietary fibre by the colon microbiome leads to the synthesis of SCFAs(butyrate, propionate, and acetate) in the gut. Butyrate ceases proliferation and induces apoptosis in colon cells [54]. It is also reported that butyrateinitiatescaspase3 activation and lead to cell death in HCT116 colon cancer cells [55]. In Caco2 cells, butyrate is reported to induce apoptosis through the mitochondrial pathway [56]. Summarizing the role of SCFA, especially butyrate, from these reports we can assume that the apoptotic effect of LS and BS fermentation supernatant is due to the presence of SCFA. BS exhibited better activity than LS which may be correlated with SCFA.

#### 3.2.9 Influence of pre-treatment with BS fermentation supernatant on differential expression of proteins in HT29 cells

Based on the above anticancer assays it was noted that the supernatant obtained from the fermentation of PIF by *B*. *bifidum* (BS) is better than that of *L*. *casei* (LS). Therefore, to discern the effect of BS on colon cancer cells HT-29, Proteomic profiling by 2D electrophoresis and protein fingerprinting was carried out.

After treatment with BS, total proteins were isolated from HT-29 cells and separated by 2D gel electrophoresis. The protein spots on the gels were scanned, and analysed using the PDQuest 8.0 software as reported earlier [25]. The up/downregulated proteins were selected and cut out physically from the gels followed by identification of proteins using peptide mass fingerprinting. As can be seen from the two dimensional map (Fig 6II), one protein is upregulated and four proteins are downregulated on treatment with BS. The details of these proteins are given in Table 1. An upregulation of apoptosis-inducing factor-mitochondria associated 1 isoform 6 (AIFM1) was observed with a fold difference of 2.315 ± 0.105. Annexin A2 isoform 2, Solute carrier family, 25 members, 35, Heat shock cognate 71 kDa protein isoform 2 and Triose phosphate isomerase isoform 3 are found to be down regulated. Among the selected downregulated proteins, significant fold change (0.514 ± 0.017) has been demonstrated by heat shock cognate 71 kDa protein isoform.

**Table 1:** Differentially expressed proteins as recognized by two dimensional electrophoresis.

	Proteins	Molecular Mass (kDa)	Isoelectric point (pI)	Fold change (treated/control)
	**Upregulated proteins**			
**P1**	Apoptosis inducing factor, mitochondria associated1 isoform 6	26.03	9.26	2.315 ± 0.105
	**Downregulated proteins**			
**P2**	Annexin A2 isoform 2	40.41	8.53	0.787 ± 0.025
**P3**	Solute carrier family 25 member 35	32.43	9.21	0.929 ± 0.018
**P4**	Heat shock cognate 71 kDa protein isoform 2	53.52	5.61	0.514 ± 0.017
**P5**	Triose phosphate isomerase isoform 3	17.958	5.39	0.579 ± .019

Apoptosis-inducing factor, mitochondria-associated 1(AIFM1) protein serves as proapoptotic as well as antiapoptotic factor. In normal cell it maintains mitochondrial respiration and redox metabolism. On the other hand, in response to the apoptotic stimuli, AIFM1 enhances DNA condensation and fragmentation, inhibits protein synthesis and triggers mitochondria to relase other apoptotic proteins. The 2D profiling demonstrated that the treatment with BS upregulates AIFM1 isoform 6 expression which can be correlated with the apoptotic effect of BS in HT29 cells. Annexin A2 is up-regulated in different tumors and controls angiogenesis, proliferation, apoptosis, cell migration, invasion, and adhesion. The *in vitro* metastatic effect of Caco-2 cells was correlated with the overexpression of Annexin A2 [57]. In the present study BS pre-treatment downregulates Annexin A2 which may be one of the reasons for the anticancer efficacy of BS.

The solute carrier family 25 member 35 (SLC25A35), a mitochondrial carrier protein facilitate transport of solutes across the inner mitochondrial membrane [58]. The membrane potential of mitochondria was dropped after the treatment of BS which might have led to the downregulation of SLC25A35 as obvious from the proteome data.

Heat shock cognate 71 kDa protein isoform 2helps in cell survival under stress conditions and the same has been reported to be overexpressed in various colon cancer cell lines [59]. Proteomic data suggest the pre-treatment with BS downregulates heat shock cognate 71 kDa protein, and thereby HT29 colon cancer cells fail to survive.

Triosephosphate isomerase converts dihydroxyacetone phosphate to glyceraldehyde 3-phosphate in glycolysis. Cancer cells particularly enhance glycolytic trail (“Warburg effect”) to meet the ATP requirement [60, 61]. Glycolytic enzymes including triose phosphate isomerase were found to be upregulated in colon cancer cells and tissues [62, 63]. BS treatment downregulates triose phosphate isomerase isoform 3 and there by prevents HT29 cells depending on the glycolytic pathway for ATP requirement.

The proteomedata of HT29 cells after exposure to BS showed that it hadgreat potential to alter the expression of different proteins which may eventually lead to the death of HT29 cells.

The *in vitro* studies carried out as discussed in the present study suggests the fermentation of the plantain inflorescence dietary fibre by selected probiotic species leading to the production SCFA that demonstrate anticancer potential by inducing apoptosis. Several randamoised blinded studies were performed in animal models to study the effect of dietary fibre against colon cancer. The anticancer effect of dietary fibre is mainly attributed to the production of SCFA in the colon, especially butyrate, due to the fermentation of dietary fibre by the beneficial microbioata in the colon [64–67]. Researchers had reported the increased colon motility in guinea pig and rats after administration of 100 mM and 5 mM butyrate respectively [68, 69]. On the other hand there are many *in vitro* reports stating the efficacy of SCFAs, butyrate in particular against colon cancer cell lines with concentration ranging upto 5 mM [70–72]. The concentration of butyrate in the fermentation supernatant we investigated for the present study falls in this range. To the best of our knowledge this is the pioneer report claiming the anticancer efficacy of fermentation supernatant of dietary fibre against colon cancer. The results from our *in vitro* experiments will definetly lay concrete basis for *in vivo* studies. The authors in their previous study has reported similar activity for the methanolic extract of plantain inflorescence against HT29 cells. The PI extract was shown to induce apoptosis, arrest cell cycle and modulate expression of pro/anti apoptotic proteins [25]. Though the anticancer properties of the dietary fibre fermentation metabolies of PI is less compared to the previously reported study, the experimental results from the earlier report and findings from the current study suggest that the consumption of PI may be helpful in maintaining better colonic biomeand hence could be a potential candidate in combating colorectal cancer.

## 4. Conclusion

**Fig 7:**
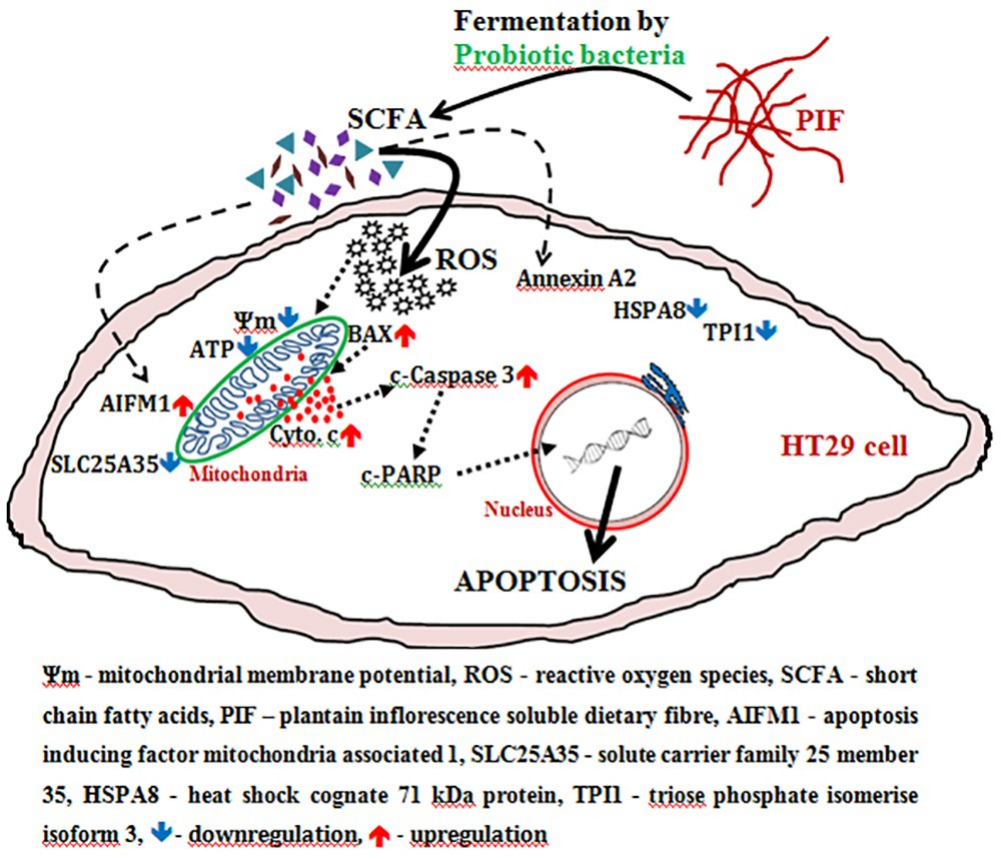
Diagrammatic representation of probableexecutionofcell death in HT29 colon cancer cells by fermentation supernatant of plantain inflorescence dietary fibre.

The soluble dietary fibre from PI exhibits potential prebiotic property as it promotes the growth of *L*. *casei* and *B*. *bifidum* which indirectly inhibits the growth of pathogenic strains and also protects the intestine. The supernatant obtained from the fermentation of PIF by *L*. *casei* and *B*. *bifidum* (LS and BS respectively) is rich in SCFAparticularly that obtained from fermentation by *B*. *bifidum*. The schematic representation of anticancer effect demonstrated by SCFA against HT29 cells, based on the experimental results, has beendescribed in Fig 7. HT29 cells treated with fermentation supernatant resulted in the over production of ROS; reduces Δψ_m_; diminishes ATP synthesis; and enhances the release ofcytochrome c. All these events ultimately leads the HT29 cells towardsapoptosis. Since BS exhibited significantly better activity, the effect of BS on protein expression level was analyzed by western blotting and 2D electrophoresis. The results showedspecified alteration in some of the proteinexpression which are known players in induction of apoptosis. The study particularlyidentified up-regulation of one of the key apoptotic inducing protein—Apoptosis-inducing factor, mitochondria-associated1. Thus it is evident from the experimental results that the fermentation supernatant contains SCFA which induces ROS mediated apoptosis in HT29 cells. In our preceding reports, it was established that the presence of bioactive phytochemicals in PI and its health potential regarding antidiabetic, cardiovascular protection and anticancer properties. As the functional food market is one of the leading food markets globally anddietaryfibremarkets is on a boom, value addition ofPI (a major part of the produce otherwise is discarded) for food, nutraceutical, and functional food application has immense socio-economicrelevance.

## Supporting information

S1 Fig Photographic image of inflorescence from *Musa paradisiaca*. (TIF)

S2 Fig Prebiotic potential of PIF determined by the change in (A) pH, (B) optical density, and (C) dry weight for *L*.*casei* and *B*. *bifidum*. Values plotted arethe average of triplicate experiments. *^,#^correspondingly showssignificantchangewhen compared to control and inulin (A-C). p ≤0.05 was considered statistically significant.
(TIF)

S3 Fig Chromatogram of standard acetic acid (1), propionic acid (2) and butyric acid (3). (TIF)

S4 Fig Photographic images showing inhibition zone against *E*. *coli* by fermentation supernatant from different experimental groups. (TIF)

S1 Table Quantification of short chain fatty acids. (DOCX)

S1 File Supporting information on methods. (DOCX)

S1 Dataset (ZIP)

## References

[1] Rattray N, Charkoftaki G, Rattray Z, Hansen JE, Vasiliou V, Johnson CH. Environmental influences in the etiology of colorectal cancer: the premise of metabolomics. Curr. Pharmacol. Rep. 2017; 3(3): 114–125. 10.1007/s40495-017-0088-z 28642837 PMC5475285

[2] Galisteo M, Duarte J, Zarzuelo A. Effects of dietary fibers on disturbances clustered in the metabolic syndrome. J Nutr Biochem. 2008; 19: 71–84. 10.1016/j.jnutbio.2007.02.009 17618108

[3] Sandler RS. Epidemiology and risk factors for colorectal cancer. Gastroenterol Clin North Am. 1996; 25: 717–735. 8960889 10.1016/s0889-8553(05)70271-5

[4] Kim Y-I. AGA technical review: Impact of dietary fiber on colon cancer occurrence. Gastroenterol. 2000; 118(6): 1235–1257. 10.1016/s0016-5085(00)70377-5 10833499

[5] Brownlee IA, Dettmar PW, Strugala V, Pearson JP. The Interaction of dietary fibres with the colon. CurrNutr Food Sci. 2006; 2:243–264.

[6] den Besten G, van Eunen K, Groen AK., Venema K, Reijngoud DJ, Bakker BM. The role of short-chain fatty acids in the interplay between diet, gut microbiota, and host energy metabolism. The J Lipid Res. 2013; 54(9): 2325–2340. 10.1194/jlr.R036012 23821742 PMC3735932

[7] Bonnet M, Buc E, Sauvanet P, Darcha C, Dubois D, Pereira B, et al Colonization of the human gut by *E*. *coli* and colorectal cancer risk, Clin Cancer Res. 2014; 20: 859–867. 10.1158/1078-0432.CCR-13-1343 24334760

[8] Ferguson LR, Harris PJ. Studies on the role of specific dietary fibres in protection against colorectal cancer. Mutat Res-Fund Mol M. 1996; 350(1): 173–184. 10.1016/0027-5107(95)00105-0 8657179

[9] Prasad KN, Bondy SC. Dietary fibers and their fermented short-chain fatty acids in prevention of human diseases. BioactCarbohydr Dietary Fibre. 2018 10.1016/j.bcdf.2018.09.001 30336163

[10] Furusawa Y, Obata Y, Fukuda S, Endo TA, Nakato G, Takahashi D, et al 2013. Commensal microbe-derived butyrate induces the differentiation of colonic regulatory T cells. Nature. 2013; 504: 446–50. 10.1038/nature12721 24226770

[11] Han R, Sun Q, Wu J, Zheng P, Zhao G. Sodium butyrate upregulates miR-203 expression to exert anti-proliferation effect on colorectal cancer cells. Cell PhysiolBiochem. 2016; 39; 1919–1929. 10.1159/000447889 27771718

[12] Christensen DP, Dahllof M, Lundh M, Rasmussen DN, Nielsen MD, Billestrup N, et al Histone deacetylase (HDAC) inhibition as a novel treatment for diabetes mellitus. Mol Med. 2011; 17: 378–390. 10.2119/molmed.2011.00021 21274504 PMC3105132

[13] Wong JM, De Souza R, Kendall CW, Emam A, Jenkins DJ. Colonic health: fermentation and short chain fatty acids. J Clin Gastroenterol. 2006; 40: 235–243. 16633129 10.1097/00004836-200603000-00015

[14] Byrne CS, Chambers ES, Alhabeeb H, Chhina N, Morrison DJ, Preston T, et al Increased colonic propionate reduces anticipatory reward responses in the human striatum to high-energy foods. Am J Clin Nutr. 2016; 104: 5–14. 10.3945/ajcn.115.126706 27169834 PMC4919527

[15] Freeland KR, Wolever TM. Acute effects of intravenous and rectal acetate on glucagon like peptide-1, peptide YY, ghrelin, adiponectin and tumour necrosis factor-alpha. Br J Nutr. 2010; 103; 460–466. 10.1017/S0007114509991863 19818198

[16] Macfarlane GT, Macfarlane S. Bacteria, colonic fermentation, and gastrointestinal health. J AOAC Int. 2012; 95(1): 50–60. 22468341 10.5740/jaoacint.sge_macfarlane

[17] Fukuda S, Toh H, Hase K, Oshima K, Nakanishi Y, Yoshimura K, et al Bifidobacteria can protect from enteropathogenic infection through production of acetate. Nature. 2011; 469(7331): 543–547. 10.1038/nature09646 21270894

[18] Jung TH, Park JH, Jeon WM, Han KS. Butyrate modulates bacterial adherence on LS174T human colorectal cells by stimulating mucin secretion and MAPK signaling pathway. Nutr Res Pract. 2015; 9(4): 343–349. 26244071 10.4162/nrp.2015.9.4.343 PMC4523476

[19] Keku TO, Dulal S, Deveaux A, Jovov B, Han X. The gastrointestinal microbiota and colorectal cancer. Am J Physiol. 2015; 308(5): G351–G363. 10.1152/ajpgi.00360.2012 PMC4346754 25540232

[20] Ríos-Covián D, Ruas-Madiedo P, Margolles A, Gueimonde M, de los Reyes-Gavilán CG, Salazar N. Intestinal short chain fatty acids and their link with diet and human health. Front Microbiol. 2016; 7: 185 10.3389/fmicb.2016.00185 26925050 PMC4756104

[21] Ivanov K, Kolev N, Tonev A, Nikolova G, Krasnaliev I, Softova E, et al Comparative analysis of prognostic significance of molecular markers of apoptosis with clinical stage and tumor differentiation in patients with colorectal cancer: a single institute experience. Hepatogastroenterol. 2009; 56: 94–98. 19453036

[22] Candela M, Turroni S, Biagi E, Carbonero F, Rampelli S, Fiorentini C, et al Inflammation and colorectal cancer, when microbiota-host mutualism breaks. World Am J Gastroenterol. 2014; 20: 908–922. 10.3748/wjg.v20.i4.908 PMC3921544 24574765

[23] Imam MZ, Akter S. *Musa paradisiaca* L. and *Musa sapientum* L.: A phytochemical and pharmacological review. J Appl Pharm Sci. 2011; 01(05): 14–20.

[24] Arun KB, Sithara T, Reshmitha TR, Akhil GC, Nisha P. Dietary fibre and phenolic-rich extracts from *Musa paradisiaca* inflorescence ameliorates type 2 diabetes and associated cardiovascular risks. J Funct Foods. 2017; 31: 198–207.

[25] Arun KB, Madhavan A, Reshmitha TR, Sithara T, Nisha P. *Musa paradisiaca* inflorescence induces human colon cancer cell death by modulating cascades of transcriptional events, Food Funct. 2018; 9: 511–524. 10.1039/c7fo01454f 29243757

[26] Guerrant GO, Lambert MA, Wayne Moss C. Analysis of short-chain acids from anaerobic bacteria by high-performance liquid chromatography. J Clin Microbiol. 1982, 16(2): 355–360. 7119103 10.1128/jcm.16.2.355-360.1982 PMC272360

[27] Pan X, Wu T, Zhang L, Song Z, Tang H, Zhao Z. *In vitro* evaluation on adherence and antimicrobial properties of a candidate probiotic *Clostridium butyricum* CB2 for farmed fish. J Appl Microbiol. 2008; 105: 1623–1629. 10.1111/j.1365-2672.2008.03885.x 18795975

[28] Sekikawa C, Kurihara H, Goto K, Takahashi K. Inhibition of β-Glucuronidase by extracts of *Chondriacrassicaulis*. Bull Fac Fish Hokkaido Univ. 2002; 53(1): 27–30.

[29] Harada K, Kawaguchi S, Supriatno, Kawashima YY, Oshida H, Mitsunobu S. S-1, an oral fluoropyrimidine anti-cancer agent, enhanced radio sensitivity in a human oral cancer cell line *in vivo* and *in vitro*: involvement possibility of inhibition of survival signal, Akt/PKB. Cancer Lett. 2005; 226: 161–168. 16134238 10.1016/j.canlet.2004.12.048

[30] Ramful D, Tarnus E, Rondeau P, Da Silva CR, Bahorun T, Bourdon E. Citrus fruit extracts reduce advanced glycation end products (AGEs)-and H_2_O_2_-induced oxidative stress in human adipocytes. J Sci Food Agric. 2010; 58(20): 11119–11129. 10.1021/jf102762s 20882960

[31] Hahn-Windgassen A, Nogueira V, Chen CC, Skeen JE, Sonenberg N, Hay N. Akt activates the mammalian target of rapamycin by regulating cellular ATP level and AMPK activity. J Biol Chem. 2005; 280: 32081–32089. 10.1074/jbc.M502876200 16027121

[32] Radhakrishnan J, Wang S, Ayoub IM, Kolarova JD, Levine RF, Gazmuri RJ. Circulating levels of cytochrome c after resuscitation from cardiac arrest: A marker of mitochondrial injury and predictor of survival. Am J Physiol. 2007; 292(2): H767–H775. 10.1152/ajpheart.00468.2006 PMC1796625 17040974

[33] Costabile A, Deaville ER, Morales AM, Gibson GR. Prebiotic Potential of a maize-based soluble fibre and impact of dose on the human gut microbiota, PLoS ONE. 2016; 11(1): e0144457 10.1371/journal.pone.0144457 26731113 PMC4701468

[34] Davis C. Enumeration of probiotic strains: Review of culture-dependent and alternative techniques to quantify viable bacteria, J Microbiol Methods. 2014, 103: 9–17. 10.1016/j.mimet.2014.04.012 24814752

[35] Byrne CS, Chambers ES, Morrison DJ, Frost G. The role of short chain fatty acids in appetite regulation and energy homeostasis. Int J Obes. 2015;39: 1331–1338. 10.1038/ijo.2015.84 PMC4564526 25971927

[36] Voltan S, Castagliuolo I, Elli M, Longo S, Brun P, D’Inca R, et al Aggregating phenotype in *Lactobacillus crispatus* determines intestinal colonization and TLR2 and TLR4 modulation in murine colonic mucosa. Clin Vaccine Immunol. 2007; 14: 1138–1148. 10.1128/CVI.00079-07 17634514 PMC2043298

[37] Collado MC, Meriluoto J, Salminen S. Adhesion and aggregation properties of probiotic and pathogen strains. Eur Food Res Technol.2008; 226: 1065–1073.

[38] Tareb R, Bernardeau M, Gueguen M, Vernoux JP. *In vitro* characterization of aggregation and adhesion properties of viable and heat killed forms of two probiotic *Lactobacillus* strains and interaction with food borne zoonotic bacteria, especially *Campylobacter jejuni*. J Med Microbiol. 2013; 62: 637–649. 10.1099/jmm.0.049965-0 23329323

[39] Reddy BS, Wynder EL. Large-bowel carcinogenesis: Fecal constituents of populations with diverse incidence rates of colon cancer. J Natl Cancer Inst. 1973; 50(6): 1437–1442. 4717561 10.1093/jnci/50.6.1437

[40] Louis P, Hold GL, Flint HJ. The gut microbiota, bacterial metabolites and colorectal cancer. Nat Rev Microbiol. 2014; 12: 661–672. 10.1038/nrmicro3344 25198138

[41] Scheppach W, Bartram HP, Richter F. Role of short chain fatty acid in the prevention of colorectal cancer. Eur J Cancer. 1995; 31A(7–8): 1077–1080. 7576995 10.1016/0959-8049(95)00165-f

[42] Bannazadeh AM, Rashtchizadeh N, Nazemieh H, Abdolalizadeh J, Mohammadnejad L, Baradaran B. Cytotoxic effects of alcoholic extract of doremaglabrum seed on cancerous cells viability. Adv Pharm Bull. (2013); 3(2): 403–408. 10.5681/apb.2013.064 24312867 PMC3848209

[43] Kumar S, Sharma VK, Yadav S, Dey S. Antiproliferative and apoptotic effects of black turtle bean extracts on human breast cancer cell line through extrinsic and intrinsic pathway. Chem Cent J. 2017; 11(1): 56 10.1186/s13065-017-0281-5 29086840 PMC5478552

[44] Borowicki A, Stein K, Scharlau D, Scheu K, Brenner-Weiss G, Obst U, et al Fermented wheat aleurone inhibits growth and induces apoptosis in human HT29 colon adenocarcinoma cells. Br J Nutr. 2010; 103: 360–369. 10.1017/S0007114509991899 19732471

[45] Wan Y, Xin Y, Zhang C, Wu D, Ding D, Tang L, et al Fermentation supernatants of *Lactobacillus delbrueckii* inhibit growth of human colon cancer cells and induce apoptosis through a caspase 3-dependent pathway. Oncol Lett. 2014; 7(5): 1738–1742. 10.3892/ol.2014.1959 24765211 PMC3997687

[46] Meshkini A, Yazdanparast R. Involvement of oxidative stress in taxol-induced apoptosis in chronic myelogenous leukemia K562 cells. Exp ToxicolPathol. 2012; 64(4): 357–365. 10.1016/j.etp.2010.09.010 21074392

[47] Domokos M, Jakus J, Szeker K, Csizinszky R, Csiko G, Neogrady Z, et al Butyrate-induced cell death and differentiation are associated with distinct patterns of ROS in HT29-derived human colon cancer cells. Dig. Dis. Sci. 2010; 55: 920–930. 10.1007/s10620-009-0820-6 19434493

[48] Heerdt BG, Houston MA, Augenlicht LH. The intrinsic mitochondrial membrane potential of colonic carcinoma cells is linked to the probability of tumor progression. Cancer Res. 2005; 65(21): 9861–9867. 10.1158/0008-5472.CAN-05-2444 16267009

[49] Shimizu S, Narita M, Tsujimoto Y. BCL2 family proteins regulate the release of apoptogenic Cytochrome C by the mitochondrial channel VDAC. Nature. 1999; 399: 483–487. 10.1038/20959 10365962

[50] Ly JD, Grubb DR, Lawen A. The mitochondrial membrane potential (Δψm) in apoptosis; an update. Apoptosis. 2003; 8: 115–128. 12766472 10.1023/a:1022945107762

[51] Chen LB, Rivers EN. Mitochondria in cancer cells, In Carney D, Sikora K, editors. Genes and Cancer, John Wiley and Sons Ltd., New York; 1990, pp. 127–135.

[52] Koehler BC, Scherr A-L, Lorenz S, Urbanik T, Kautz N, Elssner C, et al Beyond cell death–antiapoptoticBCL2proteins regulate migration and invasion of colorectal cancer cells *in vitro*. PLoS ONE. 2013;8(10): e76446 10.1371/journal.pone.0076446 24098503 PMC3789675

[53] Herceg Z, Wang ZQ. Failure of poly(ADP-ribose) polymerase cleavage by caspases leads to induction of necrosis and enhanced apoptosis. Mol Cell Bio. 1999; 19(7), 5124–5133. 10373561 10.1128/mcb.19.7.5124 PMC84355

[54] Hamer HM, Jonkers D, Venema K, Vanhoutvin S, Troost FJ, Brummer RJ. Review article: the role of butyrate on colonic function. Aliment Pharmacol Ther. 2008; 27 (2): 104–119. 10.1111/j.1365-2036.2007.03562.x 17973645

[55] Fung KMC, Brierley GV, Henderson S, Hoffmann P, McCollS R, Lockett T, et al Butyrate-induced apoptosis in hct116 colorectal cancer cells includes induction of a cell stress response. J Proteome Res.2011; 10(4): 1860–1869. 10.1021/pr1011125 21235278

[56] Ruemmele FM, Schwartz S, Seidman EG, Dionne S, Levy E Lentze MJ. Butyrate induced Caco-2 cell apoptosis is mediated via the mitochondrial pathway. Gut. 2003; 52(1): 94–100. 10.1136/gut.52.1.94 12477768 PMC1773532

[57] Xiu D, Liu L, Qiao F, Yang H, Cui L, Liu G. Annexin A2 coordinates STAT3 to regulate the invasion and migration of colorectal cancer cells in vitro. Gastroenterol ResPract. 2016; 2016: 3521453 10.1155/2016/3521453 27274723 PMC4870365

[58] Gutierrez-Aguilar M, Baines CP. Physiological and pathological roles of mitochondrial SLC25 carriers. Biochem. J. 2013; 454(3): 371–386. 10.1042/BJ20121753 23988125 PMC3806213

[59] Fan N-J, Gao J-L, Liu Y, Song W, Zhang Z-H, Gao C.-F. Label-free quantitative mass spectrometry reveals a panel of differentially expressed proteins in colorectal cancer, Biomed Res Int. 2015; 2015: 365068 10.1155/2015/365068 25699276 PMC4324820

[60] Warburg O. On the origin of cancer cells. Science.1956; 123: 309–314. 13298683 10.1126/science.123.3191.309

[61] Altenberg B, Greulich KO. Genes of glycolysis are ubiquitously overexpressed in 24 cancer classes. Genomics, 2004, 84: 1014–1020. 10.1016/j.ygeno.2004.08.010 15533718

[62] Katayama M, Nakano H, Ishiuchi A, Wu W, Oshima R, Sakuri J, et al Protein pattern difference in the colon cancer cell lines examined by two-dimensional differential in-gel electrophoresis and mass spectrometry. Surg Today. 2006; 36(2): 1085–1093. 17123137 10.1007/s00595-006-3301-y

[63] Bi X, Lin Q, Foo TW, Joshi S, You T, Shen H-M, et al Proteomic analysis of colorectal cancer reveals alterations in metabolic pathways. Mol Cell Proteomics. 2006; 5: 1119–1130. 10.1074/mcp.M500432-MCP200 16554294

[64] Perrin P, Pierre F, Patry Y, Champ M, Berreur M, Pradal G, et al Only fibres promoting a stable butyrate producing colonic ecosystem decrease the rate of aberrant crypt foci in rats. Gut. 2001; 48(1):53–61. 10.1136/gut.48.1.53 11115823 PMC1728184

[65] Govers MJ, Gannon NJ, Dunshea FR, Gibson PR, Muir JG. Wheat bran affects the site of fermentation of resistant starch and luminal indexes related to colon cancer risk: a study in pigs. Gut. 1999; 45(6):840–847. 10.1136/gut.45.6.840 10562582 PMC1727739

[66] Le Leu RK, Hu Y, Young GP. Effects of resistant starch and nonstarch polysaccharides on colonic luminal environment and genotoxin-induced apoptosis in the rat. Carcinogenesis. 2002; 23(5):713–719. 12016142 10.1093/carcin/23.5.713

[67] Bandaru SR, Yoshinobu H, Leonard AC, Barbara S, Indrane C, Chinthalapally VR. Preventive potential of wheat bran fractions against experimental colon carcinogenesis: Implications for human colon cancer prevention. Cancer Res. 2000; 60: 4792–4797. 10987288

[68] Hurst NR, Kendig DM, Murthy KS, Grider JR. The short chain fatty acids, butyrate and propionate, have differential effects on the motility of the guinea pig colon. NeurogastroenterolMotil.2014; 26(11): 1586–1596. 10.1111/nmo.12425 PMC4438679 25223619

[69] Soret R, Chevalier J, de Coppet P, Poupeau G, Derrkinderen P, Segain JP, et al Short-chain fatty acids regulate the enteric neurons and control gastrointestinal motility in rats. Gastroenterol. 2010; 138(5): 1772–1782. 10.1053/j.gastro.2010.01.053 20152836

[70] Peng L, Li ZR, Green RS, Holzman IR, Lin J. Butyrate enhances the intestinal barrier by facilitating tight junction assembly via activation of AMP-activated protein kinase in Caco-2 cell monolayers. J Nutr. 2009;139(9): 1619–25. 10.3945/jn.109.104638 19625695 PMC2728689

[71] Hatayama H, Iwashita J, Kuwajima A, Abe T. The short chain fatty acid, butyrate, stimulates MUC2 mucin production in the human colon cancer cell line, LS174T. BiochemBiophys Res Commun. 2007;356(3):599–603. 10.1016/j.bbrc.2007.03.025 17374366

[72] Xiao T, Wu S, Yan C, Zhao C, Jin H, Yan N, et al Butyrate upregulates the TLR4 expression and the phosphorylation of MAPKs and NK-κB in colon cancer cell *in vitro*. Oncol Lett. 2018; 16(4): 4439–4447. 10.3892/ol.2018.9201 30214578 PMC6126326

