# Supplementary figures and images for: Short chain fatty acids enriched fermentation metabolites of soluble dietary fibre from Musa paradisiaca drives HT29 colon cancer cells to apoptosis
Source: PLoS One. 2019 May 16;14(5):e0216604. doi: 10.1371/journal.pone.0216604 (PMC6522120; doi:10.1371/journal.pone.0216604)

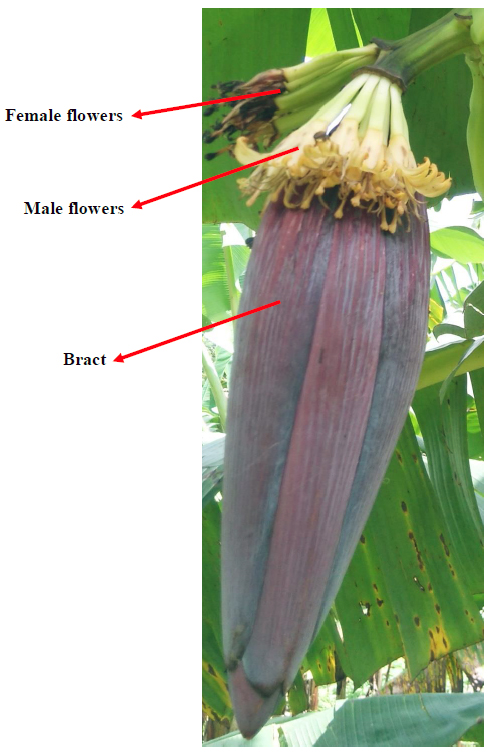

Supplement: S1 Fig — (TIF) [file pone.0216604.s001.tif]

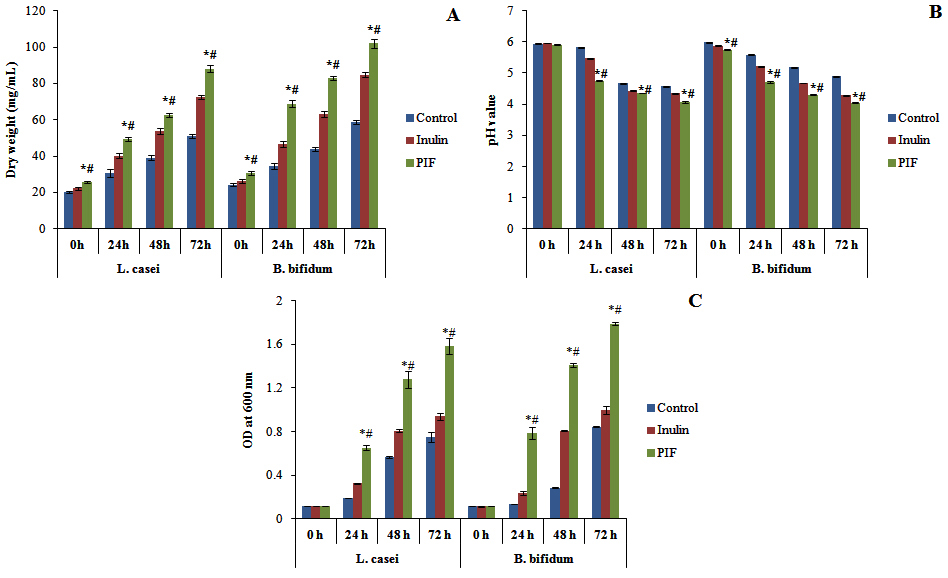

Supplement: S2 Fig — Values plotted arethe average of triplicate experiments. *,#correspondingly showssignificantchangewhen compared to control and inulin (A-C). p ≤0.05 was considered statistically significant. (TIF) [file pone.0216604.s002.tif]

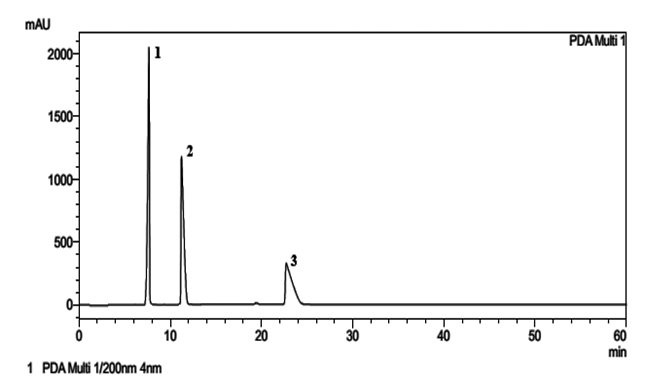

Supplement: S3 Fig — (TIF) [file pone.0216604.s003.tif]

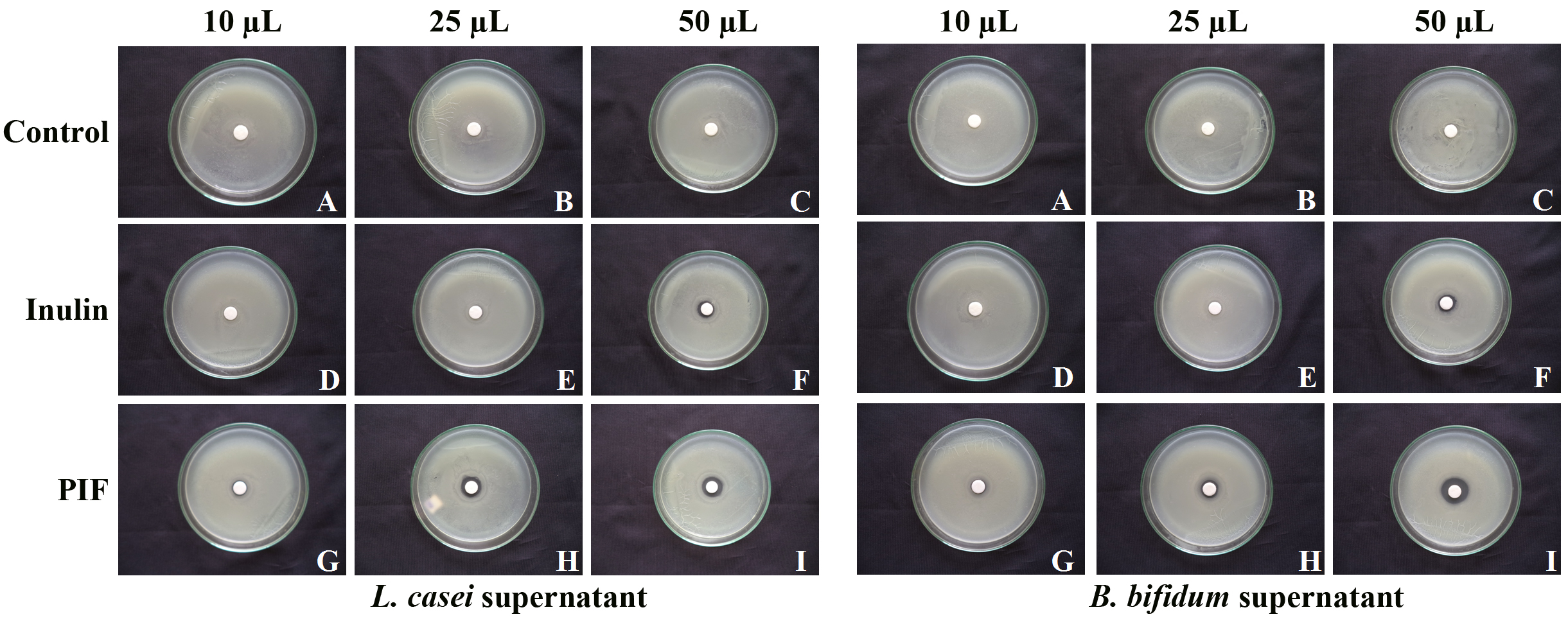

Supplement: S4 Fig — (TIF) [file pone.0216604.s004.tif]

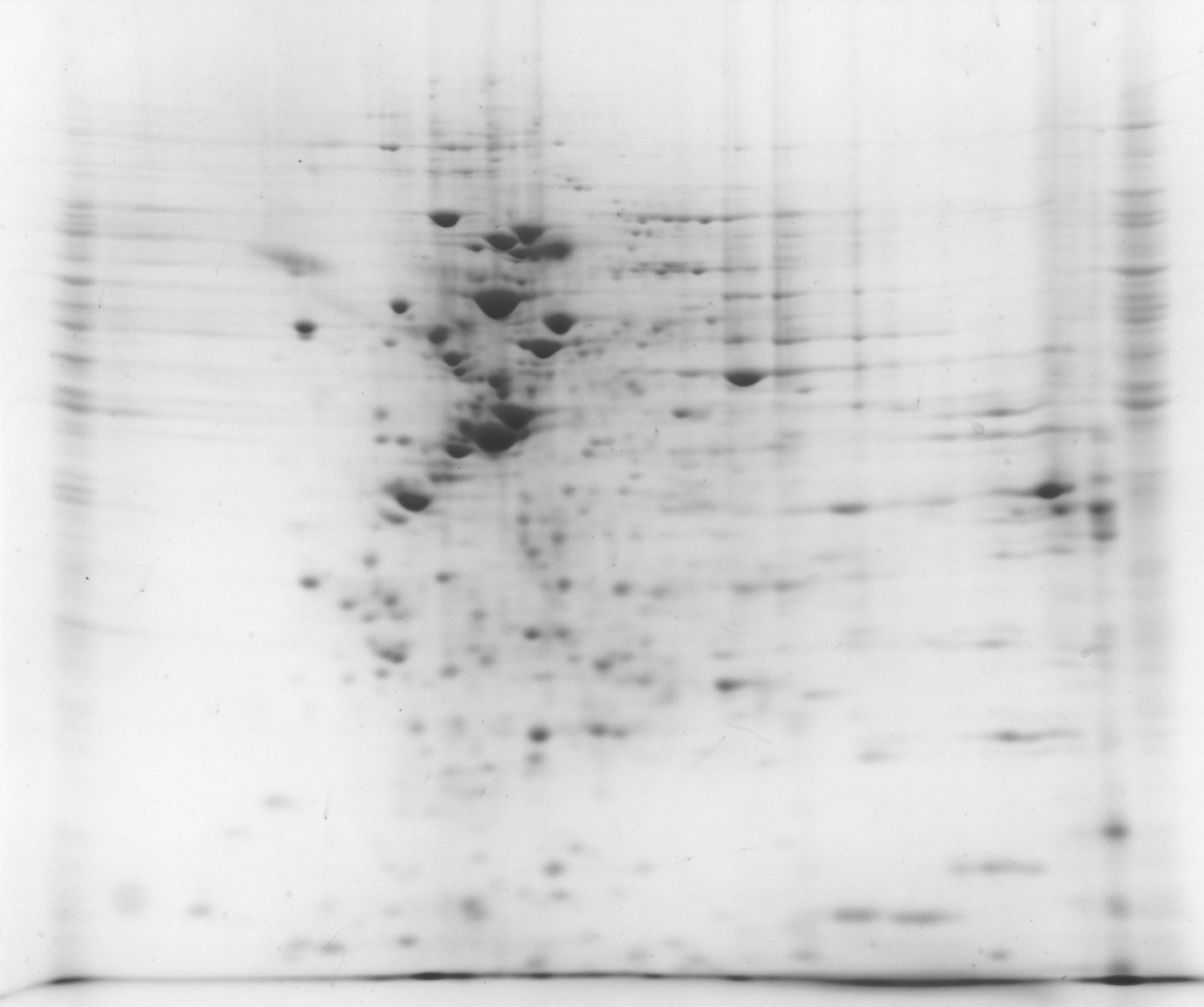

Supplement: S1 Dataset — (ZIP) [file pone.0216604.s007.zip › DATA/2D/Sample bifi (1).tif]

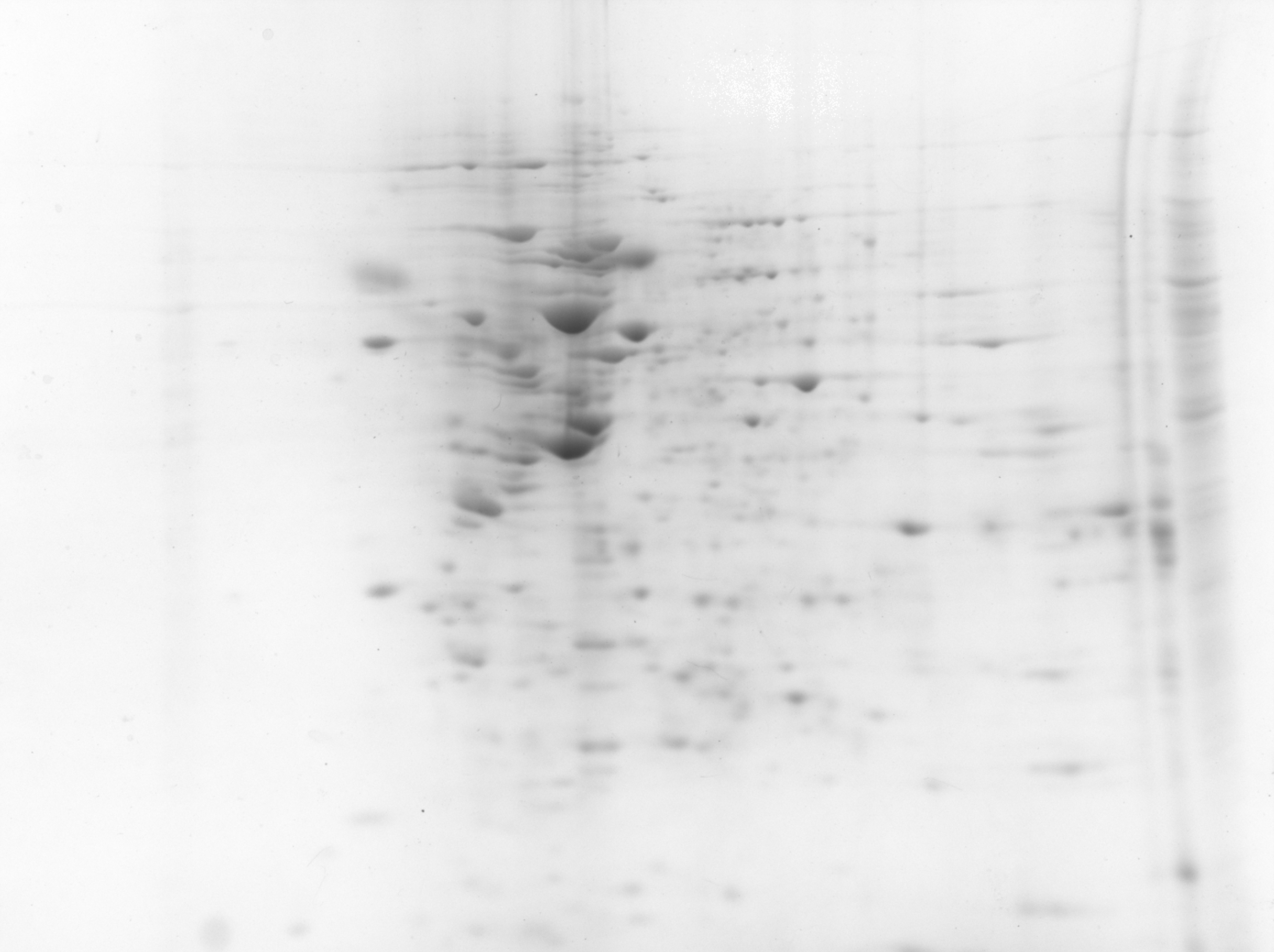

Supplement: S1 Dataset — (ZIP) [file pone.0216604.s007.zip › DATA/2D/sample bifi.jpg]

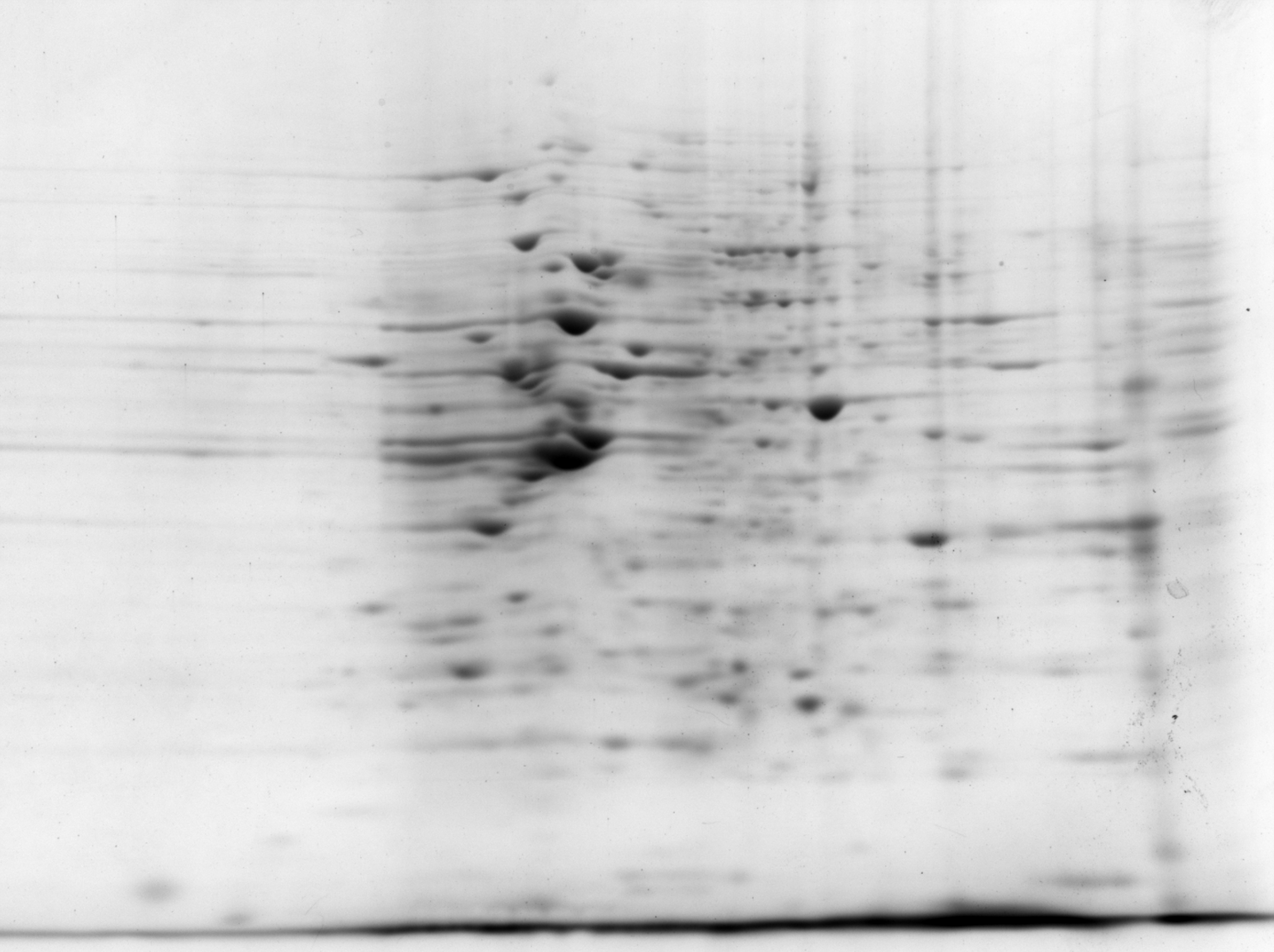

Supplement: S1 Dataset — (ZIP) [file pone.0216604.s007.zip › DATA/2D/control.tif]

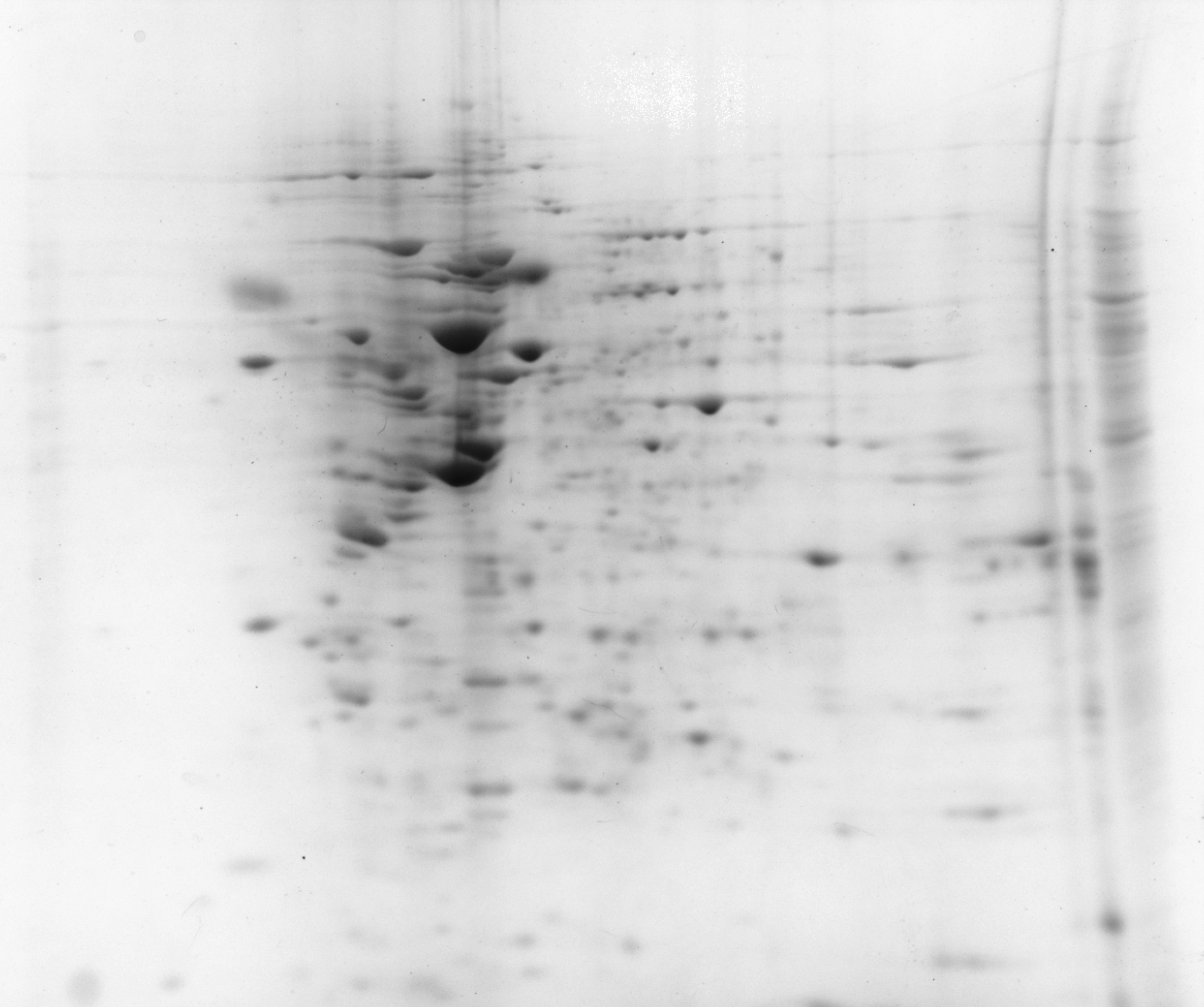

Supplement: S1 Dataset — (ZIP) [file pone.0216604.s007.zip › DATA/2D/Control bifi.tif]

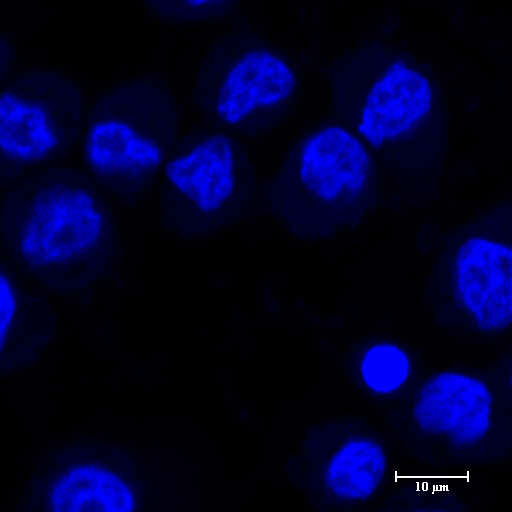

Supplement: S1 Dataset — (ZIP) [file pone.0216604.s007.zip › DATA/confocal/Positive C2 d7.jpg]

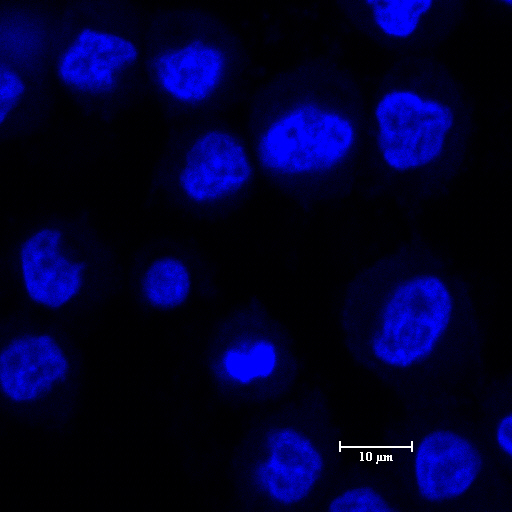

Supplement: S1 Dataset — (ZIP) [file pone.0216604.s007.zip › DATA/confocal/C3 d5.jpg]

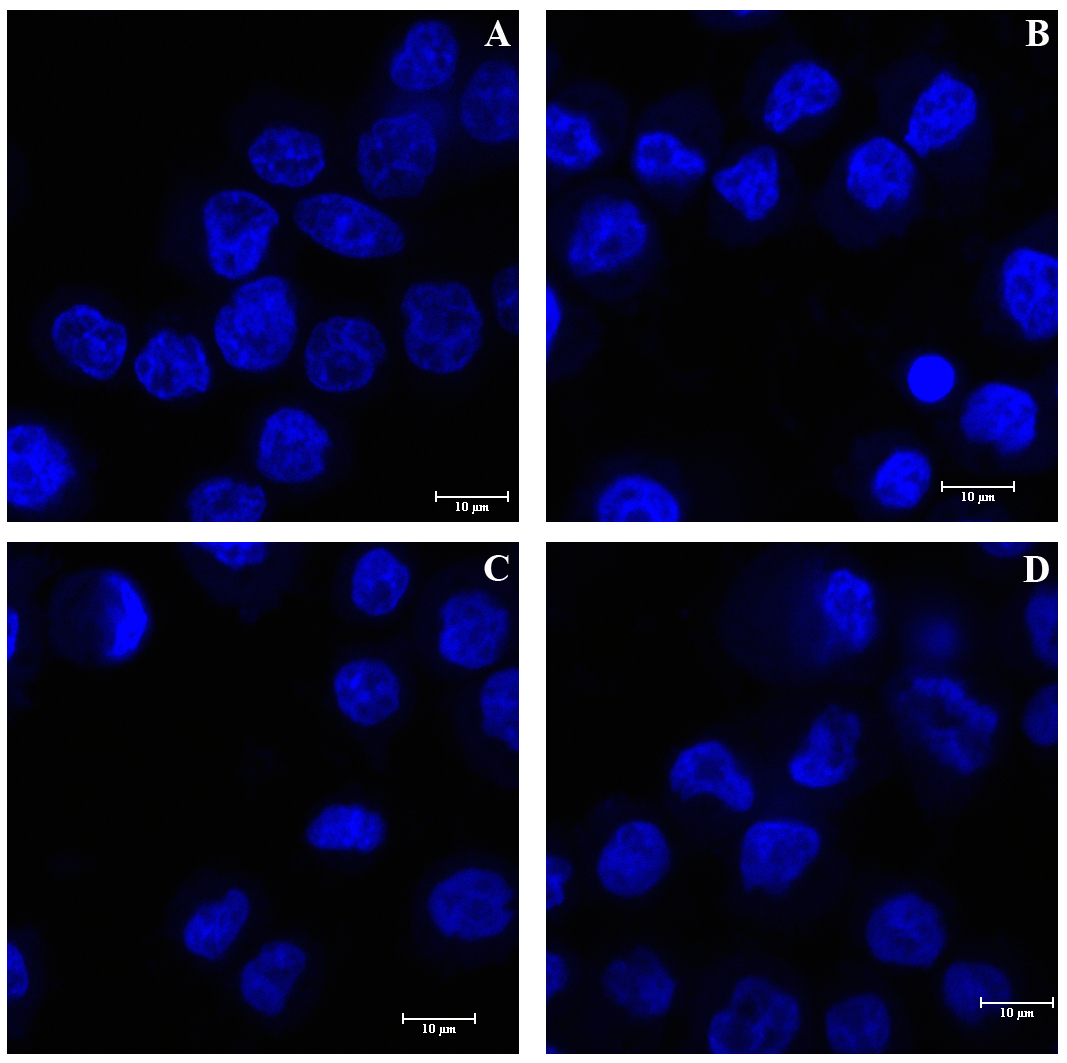

Supplement: S1 Dataset — (ZIP) [file pone.0216604.s007.zip › DATA/confocal/New folder/DNA DAM.jpg]

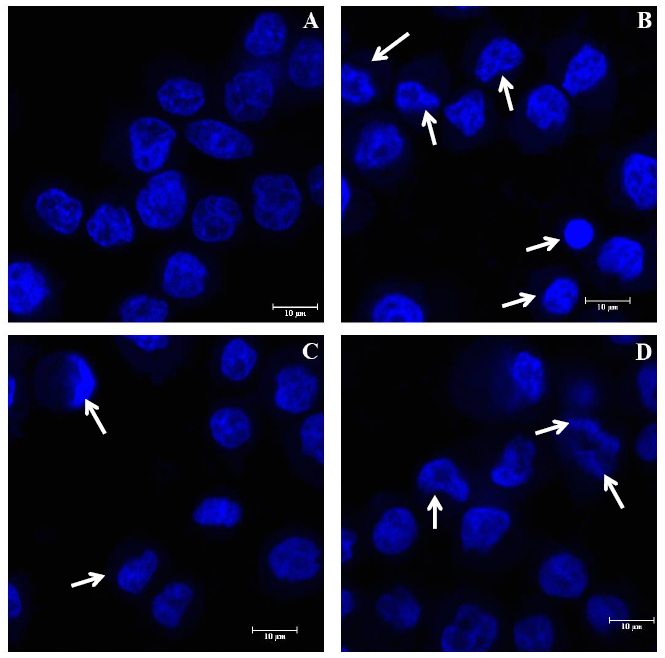

Supplement: S1 Dataset — (ZIP) [file pone.0216604.s007.zip › DATA/confocal/New folder/DNA DAMAGE FINAL TIF.tif]

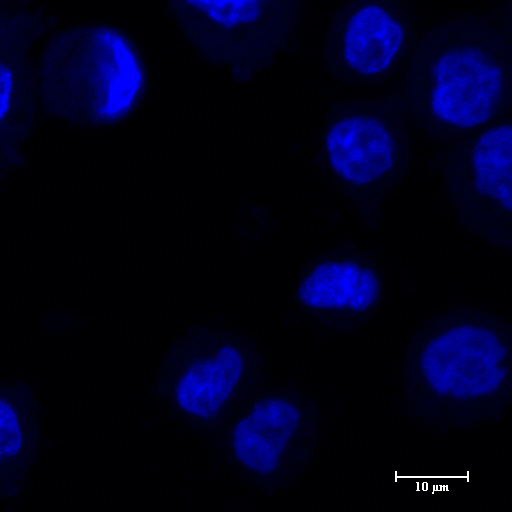

Supplement: S1 Dataset — (ZIP) [file pone.0216604.s007.zip › DATA/confocal/New folder/LS.jpg]

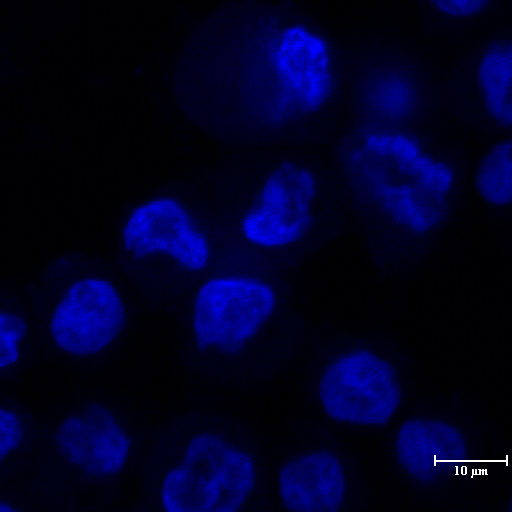

Supplement: S1 Dataset — (ZIP) [file pone.0216604.s007.zip › DATA/confocal/New folder/BS.jpg]

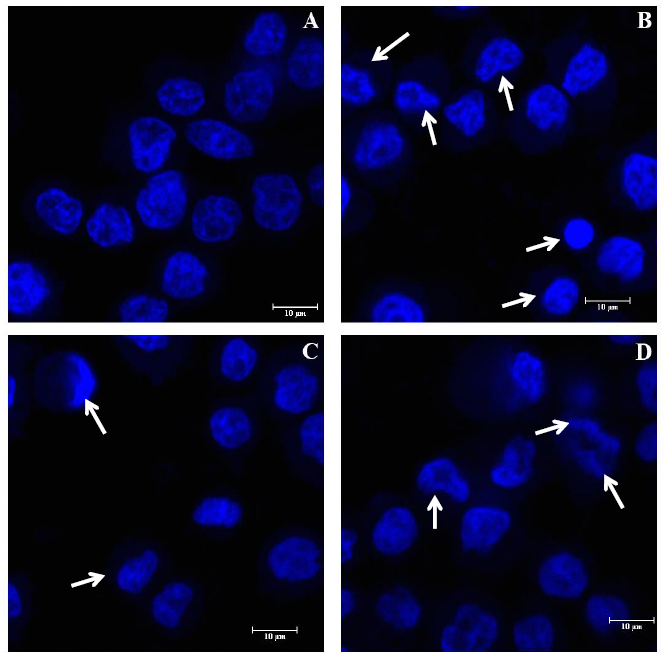

Supplement: S1 Dataset — (ZIP) [file pone.0216604.s007.zip › DATA/confocal/New folder/DNA DAM FINAL.jpg]

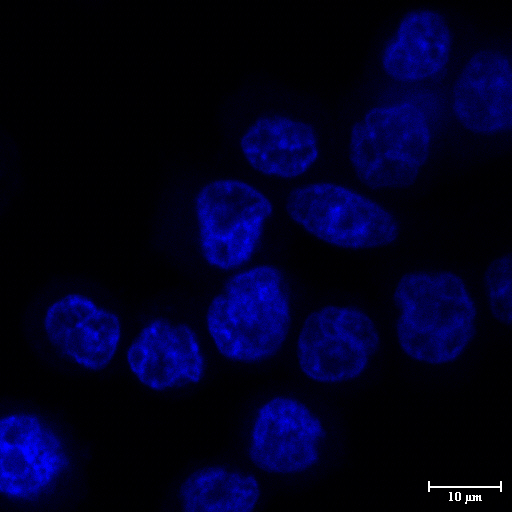

Supplement: S1 Dataset — (ZIP) [file pone.0216604.s007.zip › DATA/confocal/New folder/Control.jpg]

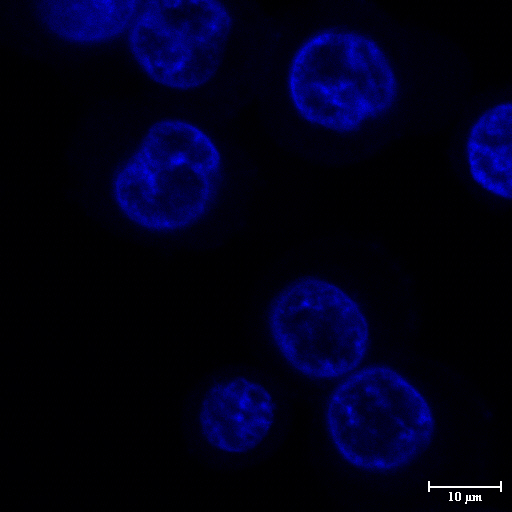

Supplement: S1 Dataset — (ZIP) [file pone.0216604.s007.zip › DATA/confocal/Control B2 d4.jpg]

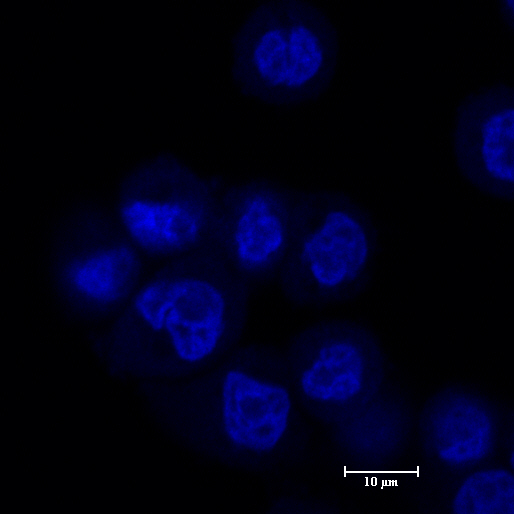

Supplement: S1 Dataset — (ZIP) [file pone.0216604.s007.zip › DATA/confocal/D3 d4.jpg]

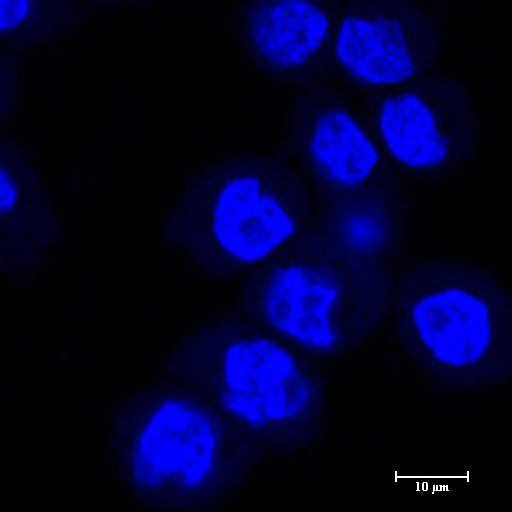

Supplement: S1 Dataset — (ZIP) [file pone.0216604.s007.zip › DATA/confocal/C3 d1.jpg]

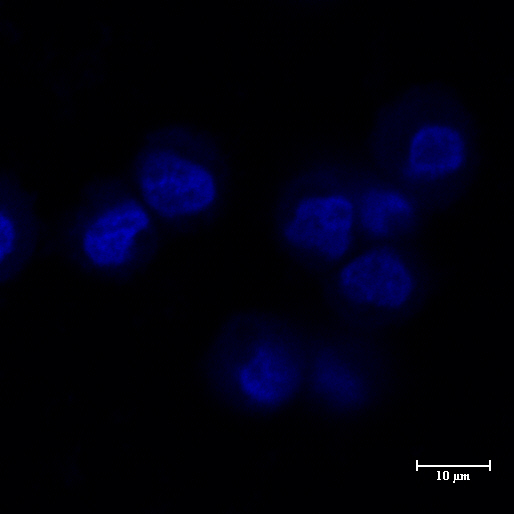

Supplement: S1 Dataset — (ZIP) [file pone.0216604.s007.zip › DATA/confocal/E2 d1.jpg]

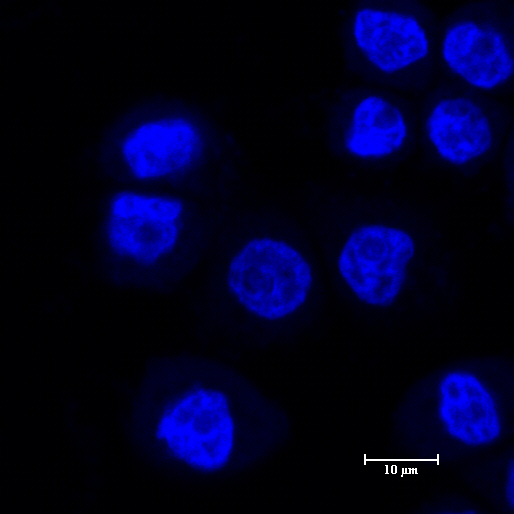

Supplement: S1 Dataset — (ZIP) [file pone.0216604.s007.zip › DATA/confocal/D2 d6.jpg]

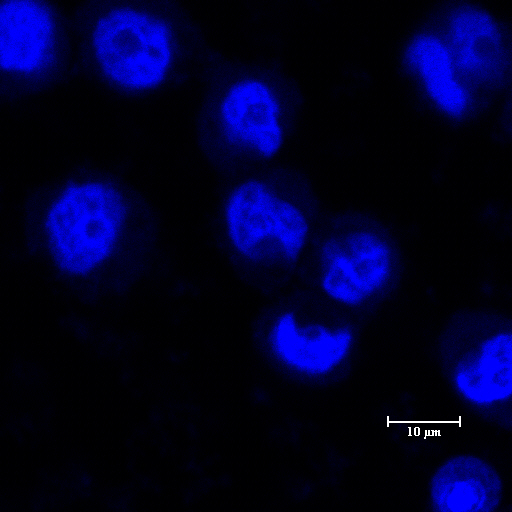

Supplement: S1 Dataset — (ZIP) [file pone.0216604.s007.zip › DATA/confocal/Positive C2 d8.jpg]
