# Supplementary material for: Short chain fatty acids enriched fermentation metabolites of soluble dietary fibre from Musa paradisiaca drives HT29 colon cancer cells to apoptosis
Source: PLoS One. 2019 May 16;14(5):e0216604. doi: 10.1371/journal.pone.0216604 (PMC6522120; doi:10.1371/journal.pone.0216604)
Supplement: S1 Table — (DOCX) [file pone.0216604.s005.docx]

**Supplementary Table 1** Quantification of short chain fatty acid

|  | *Lactobacillus casei* | | | *Bifidobacterium bifidum* | | |
| --- | --- | --- | --- | --- | --- | --- |
|  | **Control** | **Inulin** | **PIF** | **Control** | **Inulin** | **PIF** |
| AA 24 | 25.77 ± 1.52^a^ | 42.37 ± 2.16^b^ | 54.53 ± 1.48^c^ | 30.41 ± 2.11^A^ | 45.10 ± 1.49^B^ | 61.85 ± 2.32^C^ |
| AA 48 | 32.24 ± 2.72^a^ | 54.23 ± 2.55^b^ | 75.45 ± 1.16^c^ | 33.83 ± 1.36^A^ | 61.42 ± 2.54^B^ | 77.96 ± 1.16^C^ |
| AA 72 | 39.88 ± 1.42^a^ | 68.92 ± 2.85^b^ | 84.07 ± 1.63^c^ | 47.10 ± 1.24^A^ | 74.28 ± 1.44^B^ | 86.07 ± 2.13^C^ |
| PA 24 | 7.88 ± 1.77^a^ | 16.11 ± 1.46^b^ | 31.30 ± 2.40^c^ | 16.33 ± 0.91^A^ | 22.35 ± 1.16^B^ | 39.57 ± 2.32^C^ |
| PA 48 | 22.21 ± 1.05^a^ | 27.07 ± 1.89^b^ | 54.05 ± 1.11^c^ | 25.34 ± 2.47^A^ | 31.07 ± 2.04^B^ | 58.75 ± 1.27^C^ |
| PA 72 | 25.60 ± 2.37^a^ | 42.85 ± 1.41^b^ | 63.85 ± 2.56^c^ | 37.33 ± 1.55^A^ | 52.95 ± 2.31^B^ | 76.84 ± 1.74^C^ |
| BA 24 | 4.11 ± 1.20^a^ | 11.03 ± 1.09^b^ | 21.32 ± 2.14^c^ | 11.99 ± 1.04^A^ | 17.07 ± 2.18^B^ | 30.31 ± 2.63^C^ |
| BA 48 | 9.47 ± 1.34^a^ | 20.28 ± 1.24^b^ | 34.17 ± 2.33^c^ | 19.14 ± 1.57^A^ | 25.28 ± 2.14^B^ | 36.07 ± 1.24^C^ |
| BA 72 | 12.84 ± 1.12^a^ | 29.28 ± 1.75^b^ | 37.60 ± 2.12^c^ | 23.89 ± 1.40^A^ | 37.28 ± 1.47^B^ | 45.60 ± 2.15^C^ |

Quantification done at three intervals of incubation time (24 h, 48 h and 72 h). AA- Acetic acid, PA- Propionic acid, BA- Butyric acid. The results are expressed in μg/mL. Each value represents mean ± SD from triplicate measurements p≤0.05 considered significantly different. ^a,b,c^ Values with different alphabets in same row are significantly different (*Lactobacillus casei*). ^A,B,C^ Values with different alphabets in same row are significantly different (*Bifidobacterium bifidum*).
