# Supplementary material for: Short chain fatty acids enriched fermentation metabolites of soluble dietary fibre from Musa paradisiaca drives HT29 colon cancer cells to apoptosis
Source: PLoS One. 2019 May 16;14(5):e0216604. doi: 10.1371/journal.pone.0216604 (PMC6522120; doi:10.1371/journal.pone.0216604)
Supplement: S1 File — (DOCX) [file pone.0216604.s006.docx]

***Isolation of soluble dietary fire from plantain inflorescence***

Plantain inflorescence (PI), from Nendran variety, identified as Musa paradisiaca, was collected from one of the local banana farm, located at Thiruvananthapuram district of Kerala, India. Voucher specimen of Musa paradisiaca (TBGT 81481) has been deposited in the herbarium of (TBGT) of Jawaharlal Nehru Tropical Botanic Garden and Research Institute, Palode, Thiruvananthapuram, Kerala, India.

As the maturation stage changes it may affect the content of dietary fibre in inflorescence. But we look only at particular stage where the farmers used to discard the inflorescence, just after the development of fruits from the female flowers is finished. The study aims to utilize the discarded residue and to increase the value addition.

The inflorescence was washed, drained and sliced into small pieces was then freeze dried using lyophilizer (VirTis genesis, USA). The freeze dried sample was ground using a blender (Ultra centrifugal mill ZM200, Retsch, Germany) and sieved using 20 mm mesh (Vibro Sifter-PVS30, Prism Pharma Machinery, India) to obtain fine PI powder and stored in a cool dark under until further use.

Powdered sample was defatted with hexane. The soluble dietary fiber was extracted from the defatted PI powder as per the method of Bureau of Indian Standard (IS: 11062, 1984) with slight modifications. Briefly, 3 g of defatted, moisture free sample was mixed with 50 mL water and autoclaved at 120°C for 20 min. It was then cooled and the pH was adjusted to 1.5 with 5 M HCl followed by the addition of 50 mg pepsin and 200 mL of chloroform. It was incubated at 37°C for 20 h with mild stirring. After incubation the pH was adjusted to 6.0 with 3 N NaOH and 25 mL phosphate buffer, 100 mg pancreatin, 20 mg glucoamylase and few crystals of thymol were added. This mixture was incubated for 18 h at 37°C with mild stirring. After incubation the contents were centrifuged at 3000g for 30 min. To the supernatant ethanol was added in the ratio of 1:4 and centrifuged for 30 min at 3000g. The residue was collected and washed with alcohol, acetone and diethyl ether and lyophilized to obtain the soluble dietary fiber (PIF).

**Prebiotic efficacy**

For prebiotic studies 1% PIF (250 mg for 25 mL media) was added along with media and the inoculum of probiotic strains were added in such a way that 1 x 10^6^ cells/mL were the initial number of bacteria in the medium.

The stage of inflorescence, fibre and inoculum concentration will definitely play a role in the final concentration of active components of fermentation supernatant. To obtain experimental reproducibility we have strictly monitored uniformity in selection of the inflorescence stage, dietary fibre isolation, PIF concentration, bacterial number and time period for growing bacteria for various assays.
