# Supplementary material for: Short chain fatty acids enriched fermentation metabolites of soluble dietary fibre from Musa paradisiaca drives HT29 colon cancer cells to apoptosis
Source: PLoS One. 2019 May 16;14(5):e0216604. doi: 10.1371/journal.pone.0216604 (PMC6522120; doi:10.1371/journal.pone.0216604)
Supplement: S1 Dataset — (ZIP) [file pone.0216604.s007.zip › DATA/flow/control Global Sheet1_30082018162710.pdf]

FACSDiva Version 6.1.3

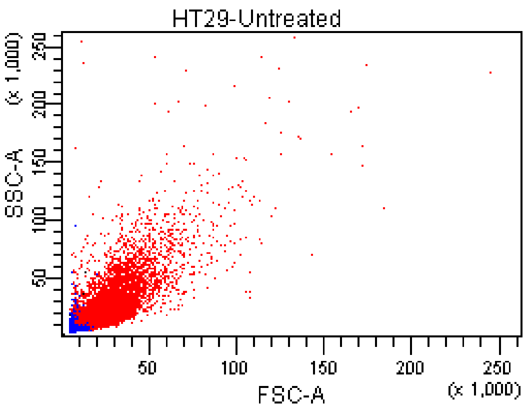

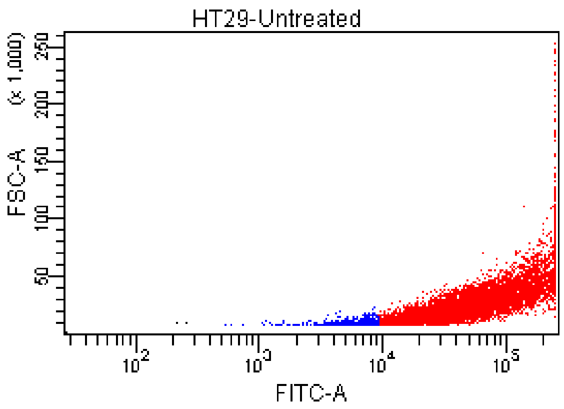

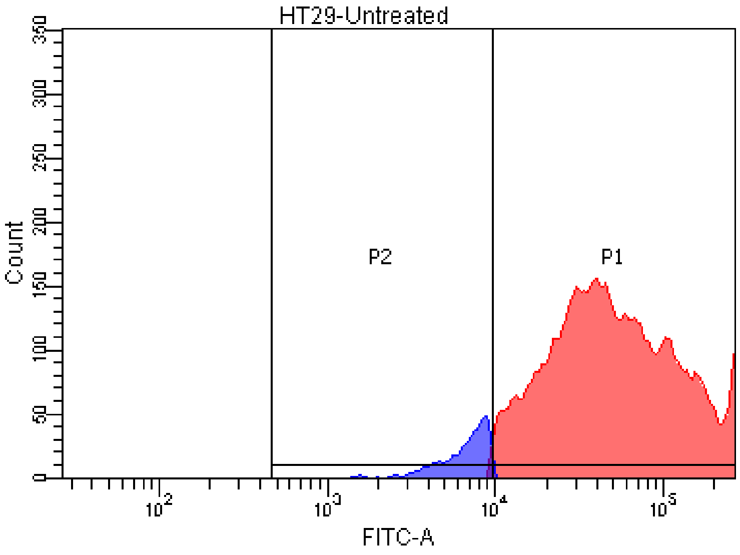

Tube: Untreated

Population	#Events	%Parent	%Total
All Events	10,000	####	100.0
P1	9,271	92.7	92.7
P2	717	7.2	7.2

Experiment Name: Mitochondria potential
Specimen Name: HT29
Tube Name: Untreated
Record Date: Aug 14, 2018 4:41:44 PM
$OP: Administrator
GUID: af5c60db-0502-493e-9943-8703d15f82d3

Population	#Events	%Parent
All Events	10,000	####
P1	9,271	92.7
P2	717	7.2
