# Supplementary material for: Short chain fatty acids enriched fermentation metabolites of soluble dietary fibre from Musa paradisiaca drives HT29 colon cancer cells to apoptosis
Source: PLoS One. 2019 May 16;14(5):e0216604. doi: 10.1371/journal.pone.0216604 (PMC6522120; doi:10.1371/journal.pone.0216604)
Supplement: S1 Dataset — (ZIP) [file pone.0216604.s007.zip › DATA/flow/Global Sheet1_14082018170938.pdf]

FACSDiva Version 6.1.3

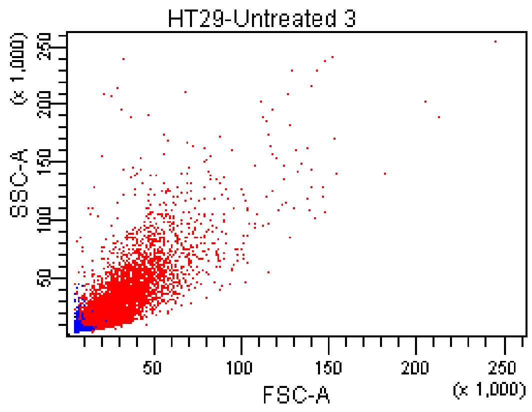

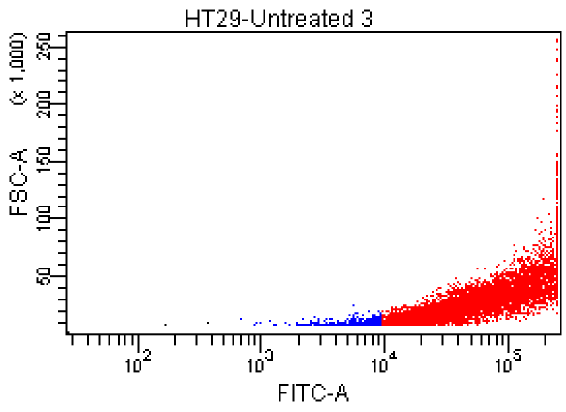

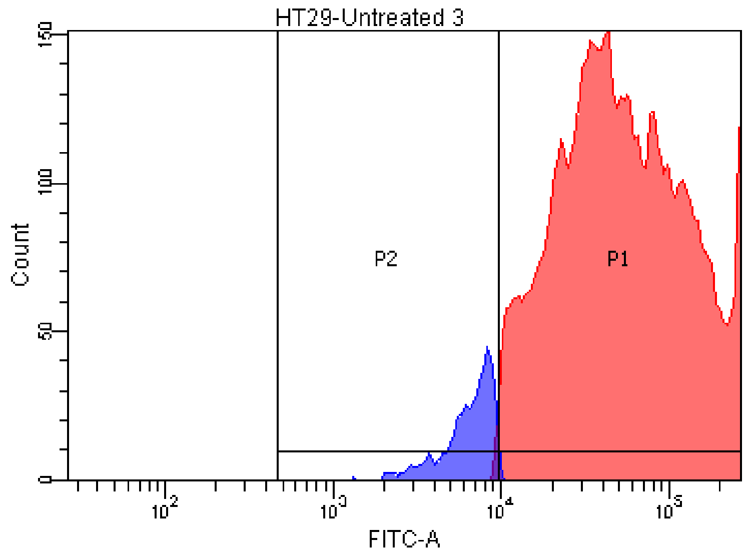

Tube: Untreated 3

Population	#Events	%Parent	%Total
All Events	10,000	####	100.0
P1	9,305	93.0	93.0
P2	685	6.9	6.9

Experiment Name: Mitochondria potential
Specimen Name: HT29
Tube Name: Untreated 3
Record Date: Aug 14, 2018 4:44:09 PM
$OP: Administrator
GUID: d6f73200-aff7-4b4a-93e1-8588a44d5f8b

Population	#Events	%Parent
All Events	10,000	####
P1	9,305	93.0
P2	685	6.9
