# Supplementary material for: Short chain fatty acids enriched fermentation metabolites of soluble dietary fibre from Musa paradisiaca drives HT29 colon cancer cells to apoptosis
Source: PLoS One. 2019 May 16;14(5):e0216604. doi: 10.1371/journal.pone.0216604 (PMC6522120; doi:10.1371/journal.pone.0216604)
Supplement: S1 Dataset — (ZIP) [file pone.0216604.s007.zip › DATA/flow/Global Sheet1_14082018171032.pdf]

FACSDiva Version 6.1.3

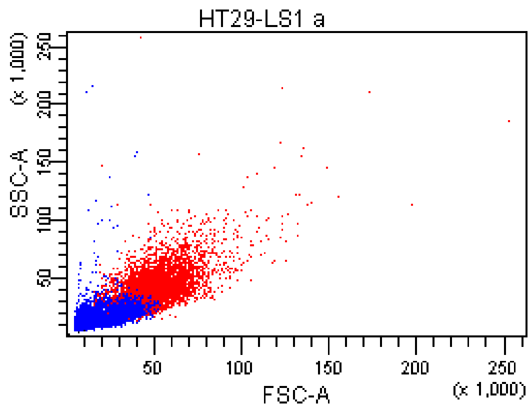

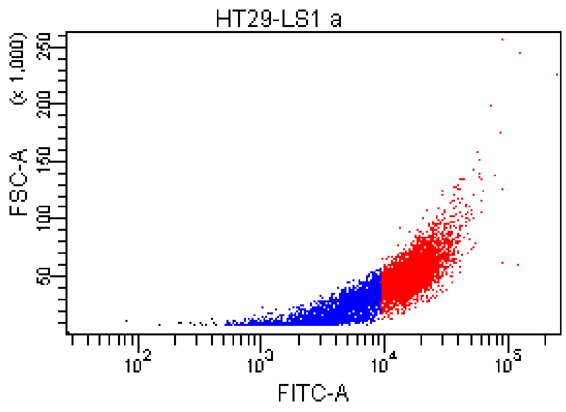

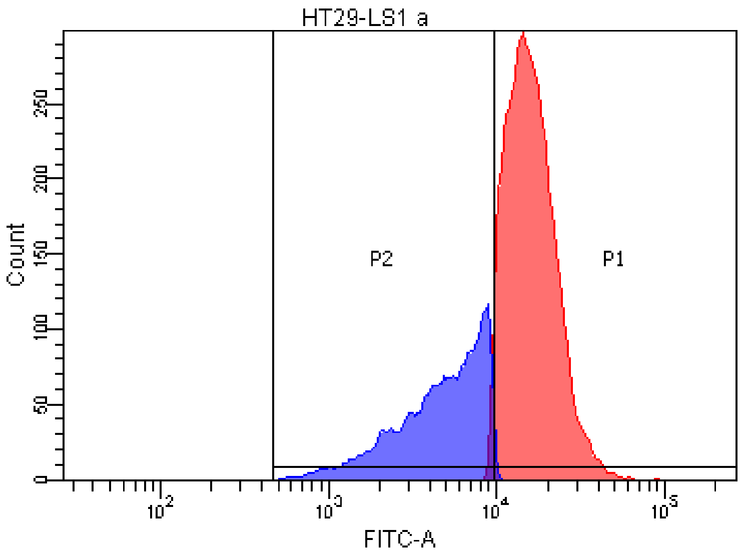

Tube: LS1 a

Population	#Events	%Parent	%Total
All Events	10,000	####	100.0
P1	6,865	68.6	68.6
P2	3,120	31.2	31.2

Experiment Name:	Mitochondria potential
Specimen Name:	HT29
Tube Name:	LS1 a
Record Date:	Aug 14, 2018 4:52:20 PM
$OP:	Administrator
GUID:	05adea4e-04a8-4236-bdea-de384a238022

Population	#Events	%Parent
All Events	10,000	####
P1	6,865	68.6
P2	3,120	31.2
