# Supplementary material for: Short chain fatty acids enriched fermentation metabolites of soluble dietary fibre from Musa paradisiaca drives HT29 colon cancer cells to apoptosis
Source: PLoS One. 2019 May 16;14(5):e0216604. doi: 10.1371/journal.pone.0216604 (PMC6522120; doi:10.1371/journal.pone.0216604)
Supplement: S1 Dataset — (ZIP) [file pone.0216604.s007.zip › DATA/flow/Global Sheet1_14082018171151.pdf]

FACSDiva Version 6.1.3

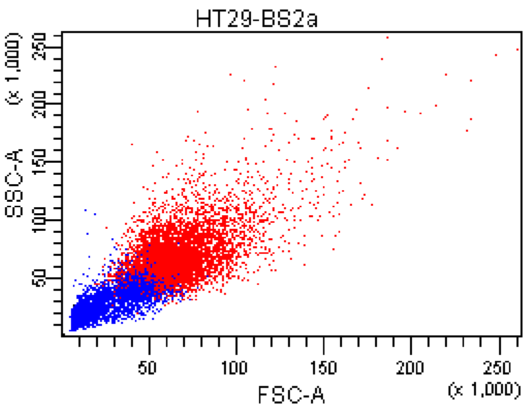

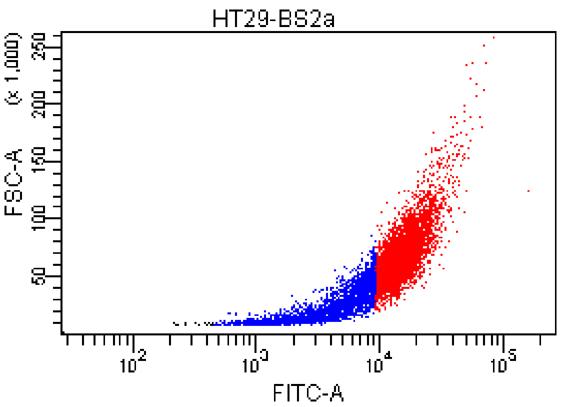

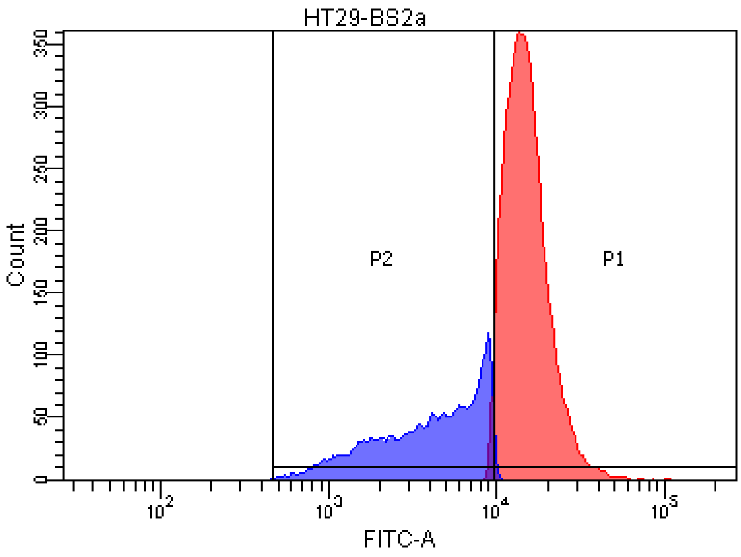

Tube: BS2a

Population	#Events	%Parent	%Total
All Events	10,000	####	100.0
P1	6,948	69.5	69.5
P2	3,036	30.4	30.4

Experiment Name: Mitochondria potential
Specimen Name: HT29
Tube Name: BS2a
Record Date: Aug 14, 2018 5:04:55 PM
$OP: Administrator
GUID: b036d508-d29c-48a7-b650-86a7f27ae66e

Population	#Events	%Parent
All Events	10,000	####
P1	6,948	69.5
P2	3,036	30.4
