# Supplementary material for: Short chain fatty acids enriched fermentation metabolites of soluble dietary fibre from Musa paradisiaca drives HT29 colon cancer cells to apoptosis
Source: PLoS One. 2019 May 16;14(5):e0216604. doi: 10.1371/journal.pone.0216604 (PMC6522120; doi:10.1371/journal.pone.0216604)
Supplement: S1 Dataset — (ZIP) [file pone.0216604.s007.zip › DATA/flow/Global Sheet1_14082018170951.pdf]

FACSDiva Version 6.1.3

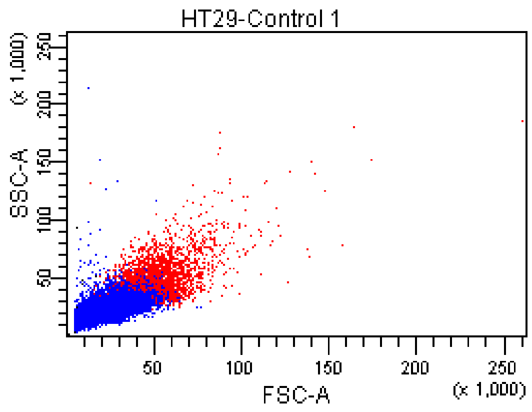

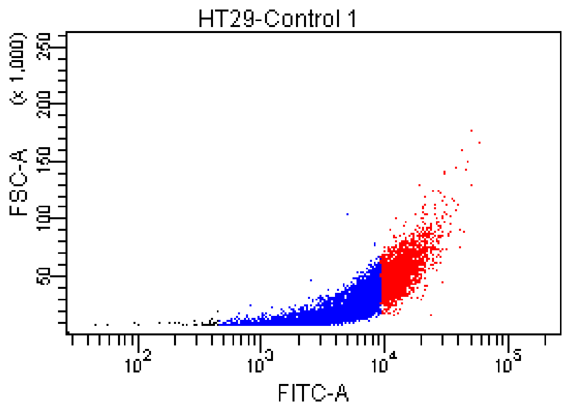

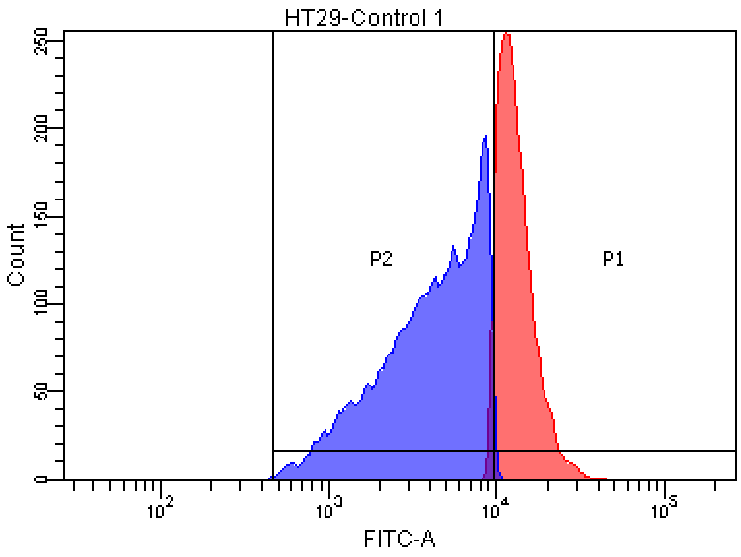

Tube: Control 1

Population	#Events	%Parent	%Total
All Events	10,000	####	100.0
P1	3,791	37.9	37.9
P2	6,171	61.7	61.7

Experiment Name: Mitochondria potential
Specimen Name: HT29
Tube Name: Control 1
Record Date: Aug 14, 2018 4:46:52 PM
$OP: Administrator
GUID: 797e4b7c-8df0-42ee-8ee4-82bf45d4cc36

Population	#Events	%Parent
All Events	10,000	####
P1	3,791	37.9
P2	6,171	61.7
