# Supplementary material for: Short chain fatty acids enriched fermentation metabolites of soluble dietary fibre from Musa paradisiaca drives HT29 colon cancer cells to apoptosis
Source: PLoS One. 2019 May 16;14(5):e0216604. doi: 10.1371/journal.pone.0216604 (PMC6522120; doi:10.1371/journal.pone.0216604)
Supplement: S1 Dataset — (ZIP) [file pone.0216604.s007.zip › DATA/flow/Global Sheet1_14082018171216.pdf]

FACSDiva Version 6.1.3

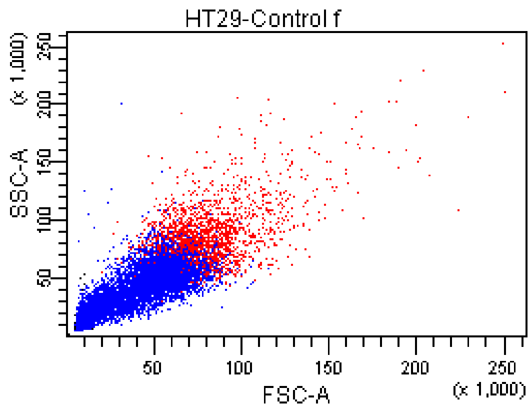

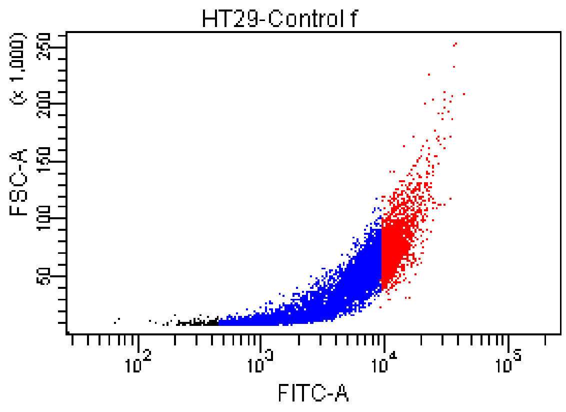

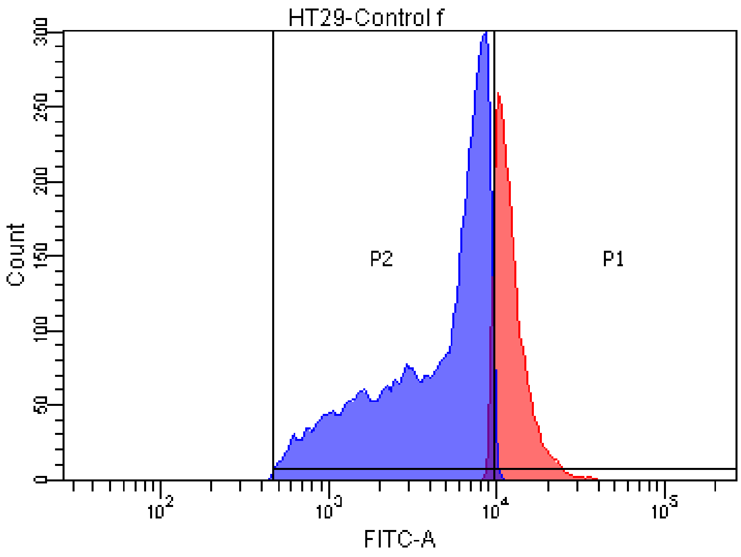

Tube: Control f

Population	#Events	%Parent	%Total
All Events	10,000	####	100.0
P1	2,811	28.1	28.1
P2	7,063	70.6	70.6

Experiment Name: Mitochondria potential
Specimen Name: HT29
Tube Name: Control f
Record Date: Aug 14, 2018 5:07:13 PM
$OP: Administrator
GUID: 31f92874-cca2-40ce-b0c9-6e6b446e30d9

Population	#Events	%Parent
All Events	10,000	####
P1	2,811	28.1
P2	7,063	70.6
