# Supplementary material for: Short chain fatty acids enriched fermentation metabolites of soluble dietary fibre from Musa paradisiaca drives HT29 colon cancer cells to apoptosis
Source: PLoS One. 2019 May 16;14(5):e0216604. doi: 10.1371/journal.pone.0216604 (PMC6522120; doi:10.1371/journal.pone.0216604)
Supplement: S1 Dataset — (ZIP) [file pone.0216604.s007.zip › DATA/flow/Global Sheet1_14082018171146.pdf]

# FACSDiva Version 6.1.3

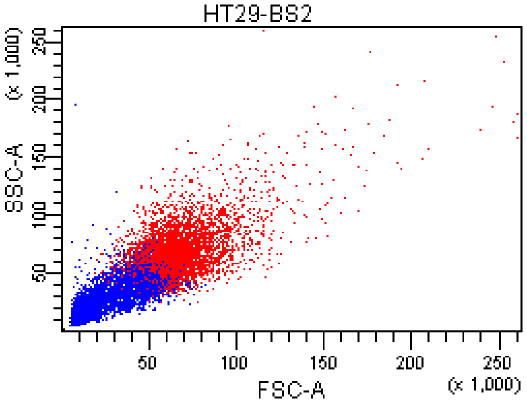


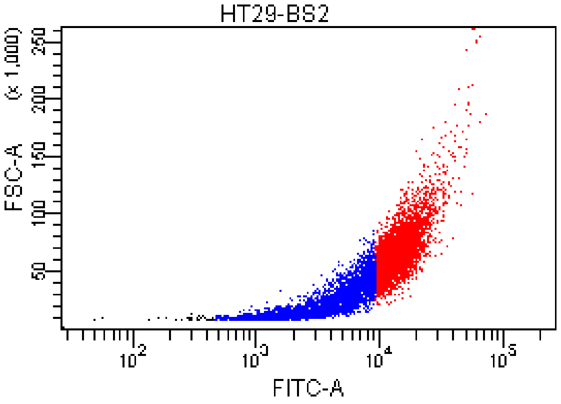


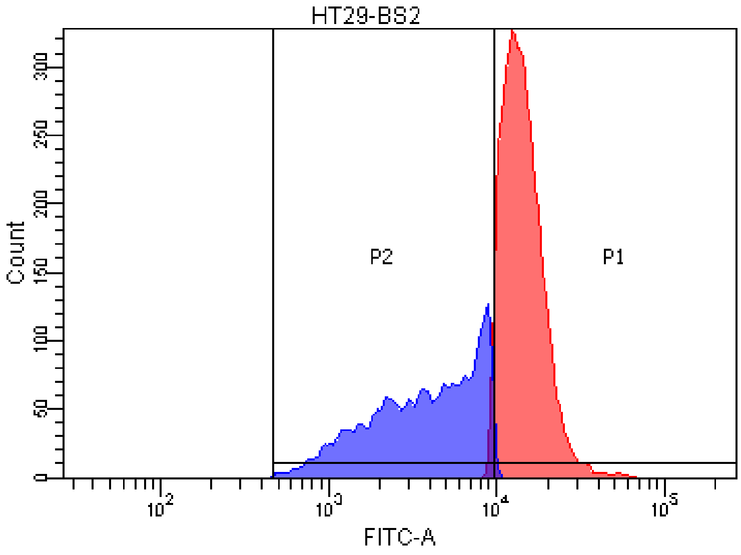


Tube: BS2

| Population | #Events | %Parent | %Total |
|---|---|---|---|
| All Events | 10,000 | #### | 100.0 |
| P1 | 5,986 | 59.9 | 59.9 |
| P2 | 3,983 | 39.8 | 39.8 |

Experiment Name: Mitochondria potential
Specimen Name: HT29
Tube Name: BS2
Record Date: Aug 14, 2018 5:04:11 PM
$OP: Administrator
GUID: b579f61e-6503-4092-9c1a-7e9101d23307

| Population | #Events | %Parent |
|---|---|---|
| All Events | 10,000 | #### |
| P1 | 5,986 | 59.9 |
| P2 | 3,983 | 39.8 |
