# Supplementary material for: Short chain fatty acids enriched fermentation metabolites of soluble dietary fibre from Musa paradisiaca drives HT29 colon cancer cells to apoptosis
Source: PLoS One. 2019 May 16;14(5):e0216604. doi: 10.1371/journal.pone.0216604 (PMC6522120; doi:10.1371/journal.pone.0216604)
Supplement: S1 Dataset — (ZIP) [file pone.0216604.s007.zip › DATA/flow/Global Sheet1_14082018171010.pdf]

FACSDiva Version 6.1.3

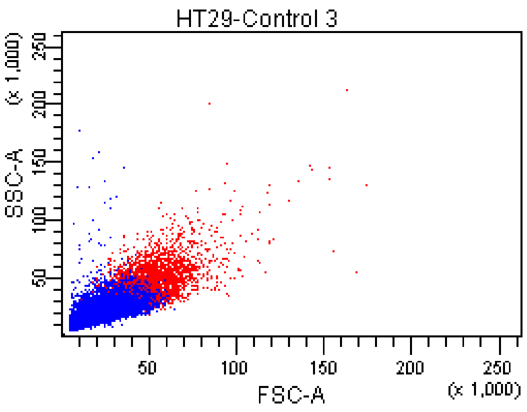

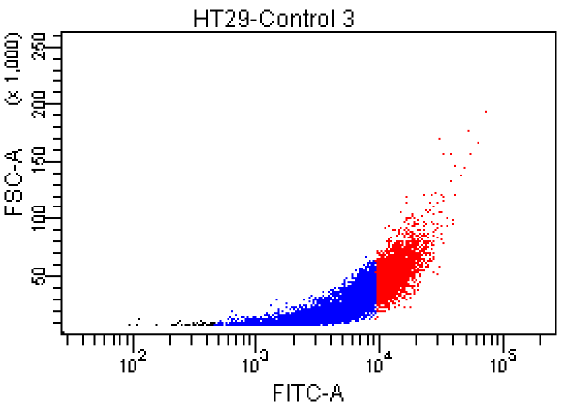

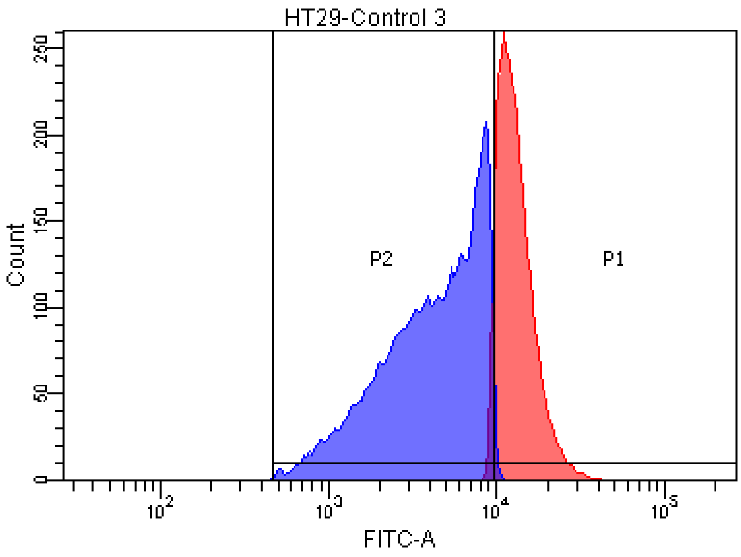

Tube: Control 3

Population	#Events	%Parent	%Total
All Events	10,000	####	100.0
P1	3,770	37.7	37.7
P2	6,186	61.9	61.9

Experiment Name: Mitochondria potential
Specimen Name: HT29
Tube Name: Control 3
Record Date: Aug 14, 2018 4:48:16 PM
$OP: Administrator
GUID: 9f19fc65-0ae3-4b3e-8433-f4fa21e24c31

Population	#Events	%Parent
All Events	10,000	####
P1	3,770	37.7
P2	6,186	61.9
